# Water-Mediated Chiral Resolution of Ag–NHC(Nucleobase) Complexes

**DOI:** 10.1021/acs.inorgchem.4c05384

**Published:** 2025-02-10

**Authors:** Alvaro Polo, Ricardo Rodríguez, Ramón Macías, Daniel Cobo Paz, Pablo J. Sanz Miguel

**Affiliations:** Departamento de Química Inorgánica, Instituto de Síntesis Química y Catálisis Homogénea (ISQCH), 16765 Universidad de Zaragoza-CSIC, Zaragoza 50009, Spain

## Abstract

This study reveals a novel role of water as a chiral inducer, demonstrating its ability to drive the asymmetric resolution of prochiral silver-nucleobase complexes. During crystallization, helical water columns spontaneously form, selectively recognizing one enantiomer of the silver complex. This enantiospecific interaction drives the separation of the *P* and *M* enantiomers, leading to the formation of enantiopure crystals, whose chirality was confirmed through X-ray crystallography.

## Graphical abstract

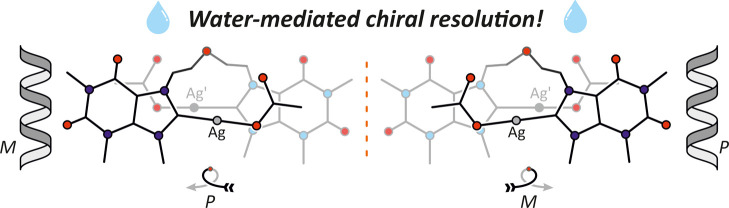

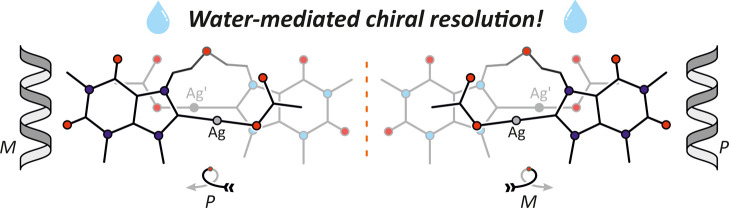

## Introduction

Water is the most abundant inorganic compound in nature, found both in the Earth’s crust and within living organisms. Hydrogen bonding,^1^ which is responsible for the primary structural cohesion between water dipoles, also drives the formation of water clusters.^2–5^ Their arrangement has been elucidated in the gaseous, liquid, and solid phases, revealing intricate geometric configurations, including discrete and polymeric constructs such as linear chains, tapes, nanotubes, layers, or three-dimensional structures.^6,7^

Water clusters typically build in the interstices of crystalline networks, filling the voids left by crystallizing molecules, thereby providing stability and, in many cases, modifying or even governing the molecular arrangement. More importantly, water clusters play a key role in many biological processes, such as protein folding, enzyme activity, or DNA stability, actively influencing their structure and properties.^8–16^ In this regard, it is expected that a water cluster assembled around a DNA fragment will adopt its inherent chirality.^17,18^ This concept can be extended to the environs of other chiral natural and artificial systems, in which chirality is transferred from an organic or metal–ligand template to a well-defined water network.^19–25^ Conversely, water clusters can also induce the assembly of supramolecular nanostructures and tune their optical properties.^26^

In recent years, the use of purine nucleobases as *N*-heterocyclic carbene (NHC) ligands has been increasingly explored.^27–29^ Most reports have focused on the antiproliferative activity of caffeine- and theophylline-based metal complexes.^30–35^ Additionally, the use of these bioinspired ligands has been expanded to catalytically^36–38^ and optically^39^ active systems as a greener and more sustainable alternative^40^ to traditional NHCs.

Our interest in metal-nucleobase complexes, led us to design and investigate flexible nucleobase- and *N*-donor based ligands.^41–45^ In this study, a bidentate ligand (**2**), in which two theophylline synthons are linked by a −CH_2_–CH_2_–O–CH_2_–CH_2_– ether group, assembles into a dinuclear silver complex (**3**) with intrinsic chirality. During the crystallization process of **3**, spontaneous formation of helical water columns occurs, in which the water selectively recognizes a homoenantiomer of complex **3** over the other. This recognition promotes the growth of enantiopure crystals and enables the chiral resolution of **3**.

## Results and Discussion

Ligand bis­[2-(7-theophyllinyl)­ethyl]­ether (**1**), was conveniently prepared in *N,N*-dimethylformamide (DMF) by reacting theophylline with bis­(2-chloroethyl)­ether (Scheme 1). Addition of water to the DMF solution prompted the precipitation of **1**, which was isolated by filtration in air. A further reaction of **1** with methyl triflate (MeOTf) allowed for the methylation of the N9 sites of the two purine bases, with the subsequent formation of the triflate salt, **2**[OTf]_2_ (Scheme 1). In order to introduce a more suitable anion (Br^–^) into the medium for the ensuing treatment with silver, NBu_4_Br was added to a solution of **2**[OTf]_2_, facilitating the isolation of **2**[Br]_2_. Finally, metalation of both C8 positions of theophylline fragments by Ag^+^ units was achieved upon treatment of **2**[Br]_2_ with silver acetate, [Ag­(OAc)], yielding [Ag_2_(OAc)_2_(**2**_–2H_)] (**3**).

**Scheme 1:**
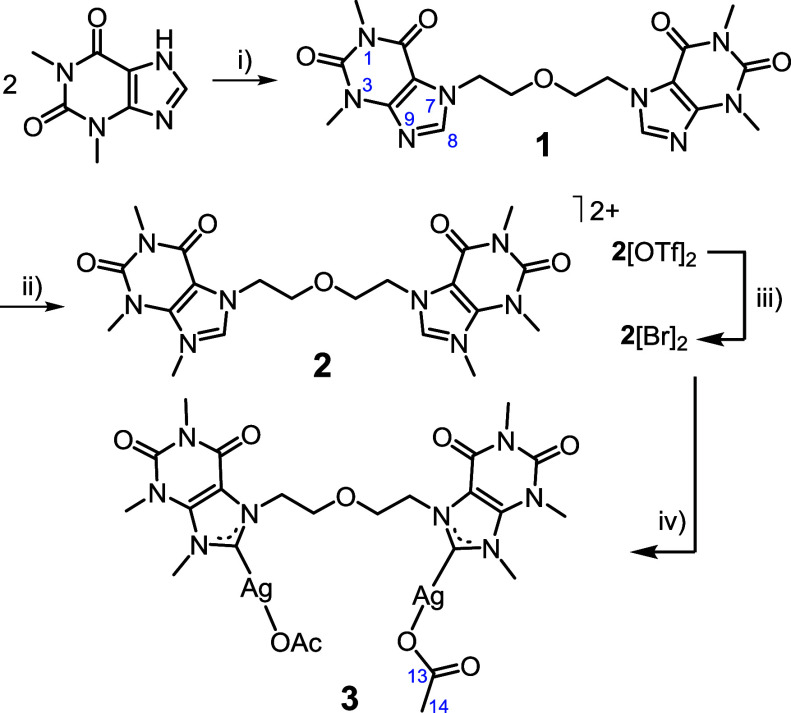
Formation of Compounds **1**, **2**[OTf]_2_, **2**[Br]_2_, and **3** ^a^

a (i) 0.5 eq bis­(chloroethyl)­ether, NaOH, DMF, 80 °C; (ii) 2 eq MeOTf, CH_2_Cl_2_, RT; (iii) [TBA]­Br, THF, RT; (iv) 4 eq [Ag­(OAc)], CH_2_Cl_2_, RT.

### NMR Analysis

The ^1^H NMR spectrum of **1** (CDCl_3_, Supporting Information) is consistent with a symmetrical (theophylline-*N*7)–CH_2_–CH_2_–O–CH_2_–CH_2_–(theophylline-*N*7) array. *N*1-CH_3_ and *N*3-CH_3_ methyl groups are observed as singlets at 3.34 and 3.55 ppm, whereas the *N*7-CH_2_ and *O*-CH_2_ signals appear at 4.37 and 3.71 ppm, respectively. Besides, the H8 proton resonates as a singlet at 7.40 ppm. Further NMR characterization is provided in the Supporting Information: ^1^H–^1^H COSY, ^13^C­{^1^H}-APT, ^1^H–^13^C HSQC, and ^1^H–^13^C HMBC spectra.

In **2**[OTf]_2_, the presence of methyl groups at the N9 sites of theophylline and the resulting positive charge, delocalized in the heterocyclic rings,^46^ provoke the expected shift of the peaks in the ^1^H NMR spectrum. This is more pronounced in those attached to the imidazolium ring, *N*7-CH_2_ (4.67 ppm) and *N*9-CH_3_ (4.14 ppm). In addition, the shift of the H8 signal (8.79 ppm) confirms methylation at the neighboring N9 position. Moreover, **2**[Br]_2_ displays a ^1^H NMR pattern similar to that of **2**[OTf]_2_, and its solubility in water reveals the H ⇋ D isotopic exchange undergone at the H8 site (Supporting Information).

The synthesis of **3** entails 2-fold C8-deprotonation of cation **2** and further coordination of both Ag^+^ ions, which is unambiguously diagnosed by the absence of the H8 signals in its ^1^H NMR spectrum (Figure 1). Further peak distribution is as follows: methyl groups from the theophylline scaffold appear as singlets at 4.27 (*N*9-CH_3_), 3.84 (*N*3-CH_3_) and 3.35 (*N*1-CH_3_) ppm, whereas those of the acetate anions (*OAc*-CH_3_) are observed at 1.90 ppm, confirming their coordination to the silver centers. Interestingly, *N*7-CH_2_ and *O*-CH_2_ resonate as broad pseudotriplets at 4.59 and 3.86 ppm. The free rotation around the sigma single bonds of the seven-membered chain, (theophylline-*N*7)–CH_2_–CH_2_–O–CH_2_–CH_2_–(theophylline-*N*7), would render each CH_2_ group chemically and magnetically equivalent, leading to pure triplets. However, the observed pseudotriplets, combined with the signal broadening, suggest hindered conformational rotation. This restriction increases the number of distinct energy states available for nuclear interactions, thus affecting the signal patterns.

**Figure 1:**
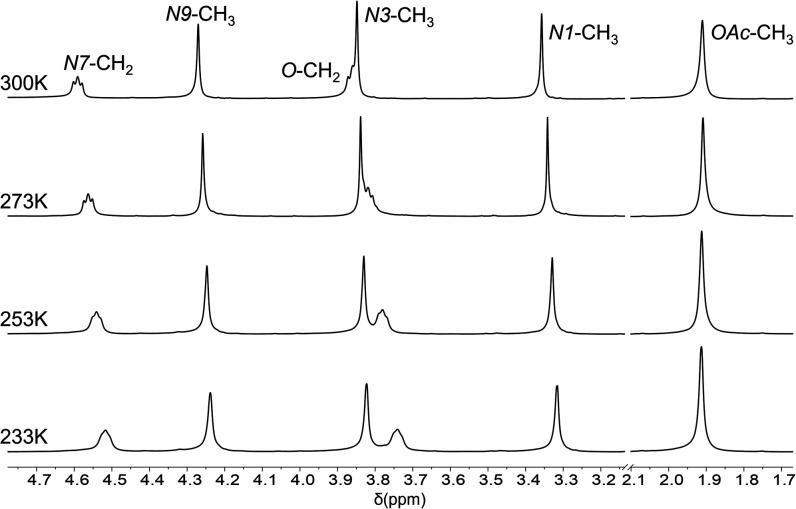
VT ^1^H NMR spectra (CD_2_Cl_2_, 400 MHz) of **3**.

This interpretation is corroborated by temperature-dependent ^1^H NMR experiments, where cooling of the samples causes further signal broadening and noticeable chemical shift changes (Figure 1). Such behavior reflects a dynamic system influenced by temperature, likely due to conformational constraints imposed by the molecular structure and/or interactions.

The ^13^C­{^1^H} NMR spectrum of **3** provides additional evidence of fluxional behavior. The C8 carbenic atom resonates as a singlet at 187.06 ppm. Besides, peaks corresponding to the acetate moiety are observed at 177.57 ppm (C13) and 22.56 ppm (C14). Typically, the presence of ^107^Ag and ^109^Ag isotopes would produce observable carbon–silver coupling doublets. However, their absence might indicate a nonrigid carbene-silver bond.^47,48^ This fluxional nature is further suggested by the broadening of the methyl acetate peak (1.90 ppm) at room temperature.

To better understand the dynamic processes, ^1^H DOSY NMR spectroscopy was employed, revealing a single species in solution with a hydrodynamic radius (*r*_H_) of 4.63 Å (*D* = 1.10 × 10^–9^ m^2^ s^–1^). This finding supports the hypothesis that any dissociation or reformation of the Ag–C bond could occur rapidly on the NMR time scale at room temperature, maintaining the observed monomeric species in solution. Together, these results emphasize the interplay between hindered rotations, fluxionality, and rapid equilibria in defining the solution behavior of the Ag-nucleobase complex **3**.

### Solid State Analysis

Compound **1** was crystallized by diffusion of diethyl ether into chloroform. After several days, single crystals of the hydrate **1**·0.25H_2_O were obtained. The partial incorporation of water into the crystal lattice was attributed to the presence of moisture in the solvents. Its solid state structure (Figure 2) is consistent with that determined by ^1^H NMR spectroscopy, where the −CH_2_–CH_2_–O–CH_2_–CH_2_– fragment is W-shaped and acts as a bridge between the two theophylline units, which are connected via their N7 sites. A notable feature of the geometry in compound **1** is that the C8–N7–C5 angles in both imidazole rings (105.86(15)°; 106.14(15)°) are larger than the corresponding C8–N9–C4 angles (103.22(15)°; 103.07(15)°), likely due to the coordination of the ether bridge at the latter positions.^46^ In addition, both purine rings are mutually tilted by 34.87(3)°.

**Figure 2:**
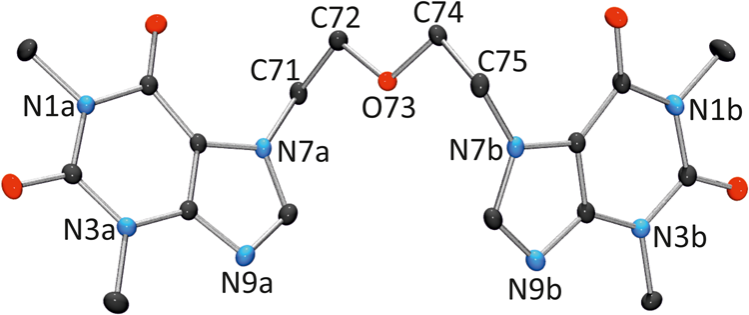
View of **1** with atom numbering scheme.

2-Fold methylation of compound **1** with methyl triflate afforded **2**[OTf]_2_, whose structure was also determined by X-ray crystallography (Figure 3). Intermolecular bond distances and angles are comparable to those of **1**, except for the endocyclic C–N–C angles within the imidazole rings, which in **2** exhibit identical values due to methylation at N9:^46^ 107.77(19)° (C8a–N7a–C5a), 107.31(19)° (C8a–N9a–C4a), 107.45(19)° (C8b–N7b–C5b), and 107.55(19)° (C8b–N9b–C4b). Folding of cation **2** in **2**[OTf]_2_ displays a slight deviation from that of **1**, with both nucleobases tilted by 11.12(10)° in a nearly antiparallel arrangement, with their C8 positions oriented toward each other.

**Figure 3:**
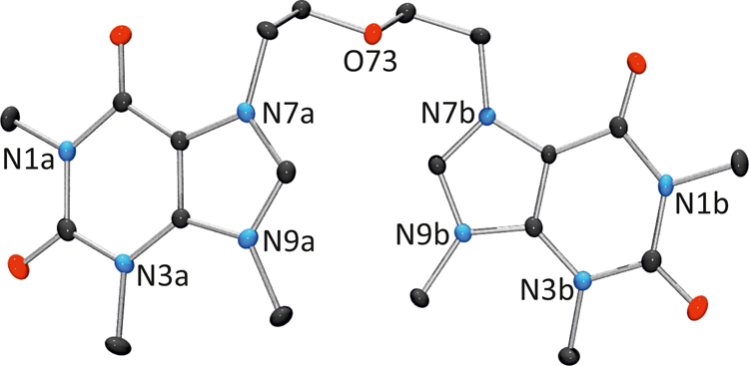
Front view of cation **2** in **2**[OTf]_2_.

Treatment of **2**[OTf]_2_ with tetrabutylammonium bromide resulted in the formation of **2**[Br]_2_, as described above. Interestingly, when **2**[Br]_2_ was crystallized in methanolic media, crystals of the **2**[Br]_2_·CH_3_OH adduct were obtained. Although interatomic distances and angles of cation **2** in **2**[Br]_2_·CH_3_OH are almost identical to those in **2**[OTf]_2_, its conformation differs. As depicted in Figure 4, cation **2** exhibits a U-conformation (*C*_s_ symmetry), accommodating a Br^–^ anion and a methanol molecule in its cavity, forming a host–guest system, in which Br^–^ anion is connected by hydrogen bonding to methanol (MeOH···Br, 3.250(3) Å), and attached to the cationic host via electrostatics and anion−π interactions.

**Figure 4:**
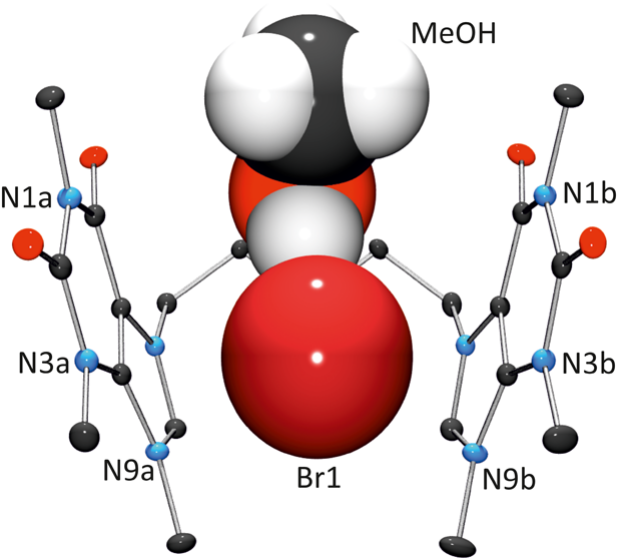
Detail of the host–guest system {Br,MeOH ⊂ **2**} in **2**[Br]_2_·CH_3_OH. A Br^–^ anion has been omitted for clarity.

To use cation **2** as an NHC ligand precursor, the C8 position of the nucleobase was deprotonated by adding four equivalents of silver acetate to a suspension of **2**[Br]_2_ in dichloromethane. As anticipated above, this treatment resulted in the coordination of a silver ion to each nucleobase via its C8 site, with the silver linear coordination sphere completed by an acetate ligand, forming [Ag_2_(OAc)_2_(**2**_–2H_)] (**3**). Compound **3** was initially isolated as a white, solvent-free powder. However, crystallization from a dimethyl sulfoxide (DMSO) solution yielded crystals that contained two solvent molecules in the asymmetric unit, identified as **3**·2DMSO.

Figure 5 provides a view of one enantiomer (see below) of **3** in **3**·2DMSO. The *C*_2_ symmetry observed in NMR measurements is disrupted in the crystal structure. As a result, while the coordination environments of silver are analogous, they are not identical in the solid state. Bond distances involving Ag^+^ ions are within the expected range: 2.083(3) Å (Ag1–C8a), 2.137(2) Å (Ag1–O11), 2.081(3) Å (Ag2–C8b), and 2.156(2) Å (Ag2–O21). In addition, separation between the two silver atoms is 3.9538(4) Å, which is probably too long to be considered a metallophilic interaction.^49^ Coordination angles involving silver atoms deviate slightly from linearity: C8a–Ag1–O11, 170.78(10)°; (C8b–Ag2–O21), 170.42(10)°. Regarding its spatial conformation, complex **3** adopts an antiparallel orientation, similar to that of **2**[OTf]_2_, with the C8 positions facing each other and a mutual tilt angle of 20.00(6)°. The acetate ligands are twisted relative to the nucleobases attached to the same silver atom, with torsion angles of 23.10(13)° (Ag1) and 34.85(16)° (Ag2). The uncoordinated oxygen atoms (O12, O22) point toward the Hoogsteen edges of the respective nucleobases.

**Figure 5:**
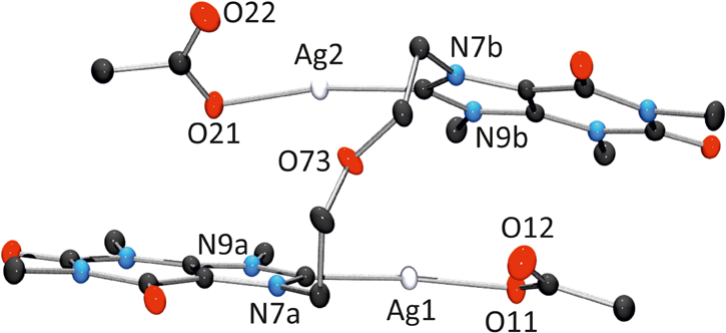
Side view of the (*P*)-**3** enantiomer in **3**·2DMSO. For *P*- and *M*-chirality see Figure 6.

More importantly, **3** crystallizes in two chiral isomers (Figure 6), *P* and *M*, both of which are present in the crystal packing of **3**·2DMSO. Both forms exhibit nearly identical (enantiomeric) arrangements, with the chirality of **3** in the solid state arising from its three-dimensional folding. To describe the two enantiomers of **3**, namely (*P*)-**3** and (*M*)-**3**, a scheme analogous to that employed for helicenes is utilized. As depicted in Figure 6 (left), a *P*-loop can be described starting at front-right acetate ligand, passing through its coordinated Ag and the adjoining nucleobase, then turning clockwise via the ether bridge to the other nucleobase, before reaching Ag′ and its attached back-left acetate ligand. A *M*-loop follows a counterclockwise rotation (Figure 6, right).

**Figure 6:**
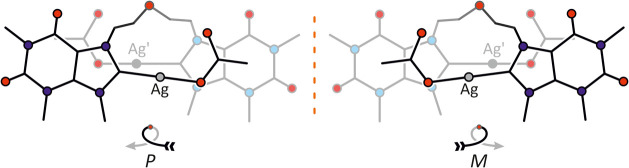
Conceptual depiction of the helical chirality exhibited by the (*P*)-**3** and (*M*)-**3** enantiomers in **3**·2DMSO.

Remarkably, the crystal packing of the racemic solvate **3**·2DMSO exhibits a staggered arrangement, with π–π stacking interactions between symmetry-related nucleobases in an ···*M*···*P*··· pattern (*M* and *P* refer to the different enantiomers of **3**), with stack distances of 3.4 Å (Figure 7).

**Figure 7:**
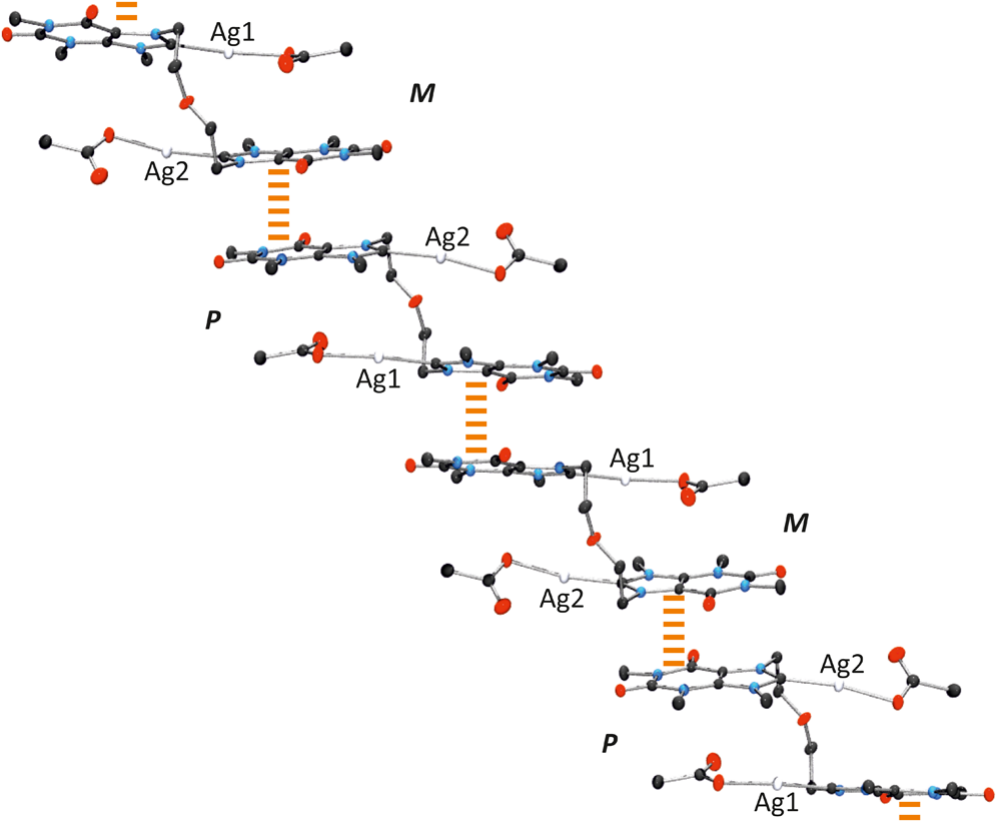
Staggered arrangement of **3** in **3**·2DMSO featuring π–π stacking. For *P*- and *M*-chirality see Figure 6.

Further crystallization assays were carried out by diffusing hexane into solutions of **3** in either dichloromethane or chloroform. Here, the use of undried solvents (taken directly from the bottle) resulted in the formation of crystalline hydrates with the formula **3**·3H_2_O. Thus, during the crystallization process, trace amounts of water are extracted from solvents, resulting in the formation of stable, durable crystals. Once the crystals have formed and the solvents are removed, they remain stable without any water loss for weeks when stored in air at room temperature.

Under the microscope, the resulting crystals appeared indistinguishable, differing only slightly in size and other expected characteristics. To determine the chirality of the crystals, they were manually selected and mounted onto a diffractometer for characterization. The first crystal selected for X-ray diffraction analysis was assigned to the trigonal *P*3_1_21 space group, and further identified as (*M*)-**3**·(*P*)-3H_2_O, where *M* indicates the helicity of **3** (Figure 6) and *P* the helicity of the water chain (see below). Its absolute configuration was unambiguously established based on the Flack parameter: 0.029(8). In order to verify whether all the crystalline material exhibited the same chirality, several arbitrarily selected samples were analyzed. After multiple attempts, including X-ray measurements and refinements, we successfully identified a crystal that fitted within the *P*3_2_21 space group. The Flack parameter of 0.017(9) confirmed it as (*P*)-**3**·(*M*)-3H_2_O, which is the enantiomer of (*M*)-**3**·(*P*)-3H_2_O. Subsequent measurements of other picked crystals showed an equal distribution of the two enantiomers.

Hereinafter, only the crystal structure of (*M*)-**3**·(*P*)-3H_2_O is described, as it is almost identical to that of (*P*)-**3**·(*M*)-3H_2_O. In (*M*)-**3**·(*P*)-3H_2_O, complex **3** exhibits a crystallographic *C*_2_ axis that intersects the oxygen atom of the bridging ether, making the two halves of **3** symmetry-equivalent. Molecular arrangement of complex **3** in (*M*)-**3**·(*P*)-3H_2_O is similar to that exhibited in **3**·2DMSO. However, while in the DMSO adduct the terminal oxygens of the acetate ligands are oriented toward the ether chain (Figure 6), in (*M*)-**3**·(*P*)-3H_2_O and (*P*)-**3**·(*M*)-3H_2_O, they are rotated by 180°, pointing instead toward sugar edges of the theophylline fragments (Supporting Information).

The unit cell of (*M*)-**3**·(*P*)-3H_2_O contains complex **3** and two crystallographically different water molecules (O1w and O2w), both of which participate in hydrogen bonding, interacting with each other and/or with the acetate ligands. Hydrogen bonding distances are as follows: O1w···O1w′, 2.78(4) Å; O1w···O2w, 2.767(18) Å; O2w···O2w′, 2.878(16) Å; O1w···O12­(acetate), 2.641(19) Å. In the crystal packing, the hydrogen bonding network between H_2_O molecules organizes into helical, enantiopure one-dimensional water chains. Specifically, symmetry related O2w water molecules form a *P*-helix that twists around the *c* axis (Figure 8).

**Figure 8:**
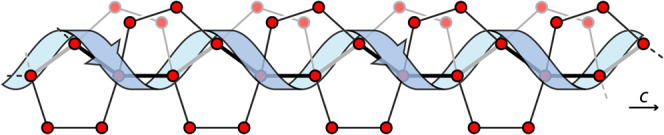
Schematic representation of the *P*-helical backbone within the water cluster of (*M*)-**3**·(*P*)-3H_2_O. Red dot: oxygen atom.

The stability of this homochiral backbone is reinforced by envelope-like 5-membered water clusters, which surround the helix, and consist of three consecutive water molecules within the *P*-helix (O2w), along with two additional water molecules (O1w). The resulting fused pentagons form an intricate 1D array that preserves the *P*-helicity (Figure 8).

In addition, the cisoid arrangement of both acetate terminal oxygens toward the sugar edges allows complex **3** to bind the water cluster via 2-fold O1w···O12 (acetate) hydrogen bonds (Figure 9). These interactions are shorter than those involving water–water contacts (see above).

**Figure 9:**
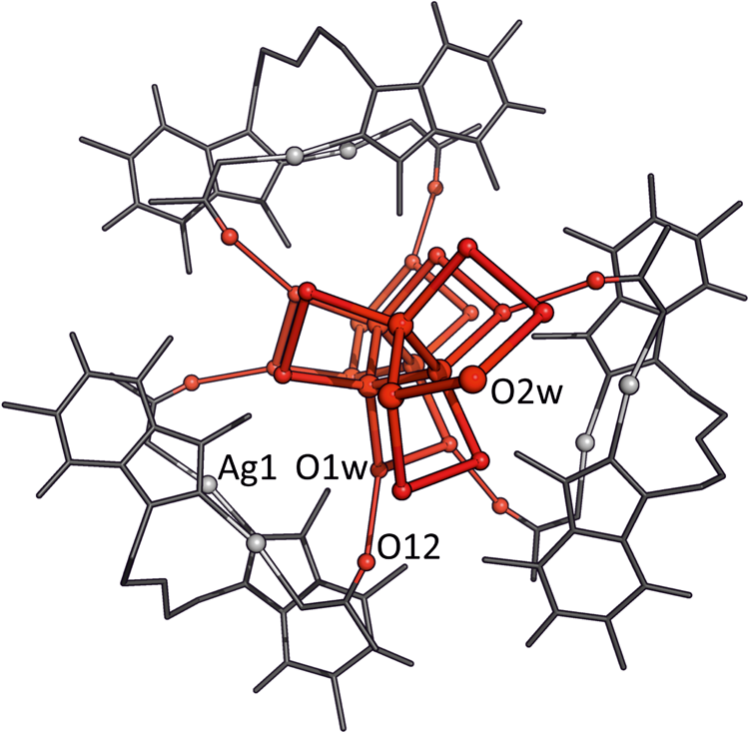
Detail of the helical arrangement of **3** in (*M*)-**3**·(*P*)-3H_2_O around the respective chiral water chains.

Remarkably, the helical arrangement of the water array induces an enantiospecific hydrogen bonding interaction with complex **3**, resulting in spontaneous chiral resolution. Such enantiomeric discrimination is not observed in the DMSO adduct. In particular, left-handed (*M*)-**3** units are attached to the right-handed (*P*) water chains to form (*M*)-**3**·(*P*)-3H_2_O. In the case of the (*P*)-**3**·(*M*)-3H_2_O, the contrary occurs, and right-handed complex (*P*)-**3** is recognized by the left-handed (*M*) water helix. Besides, positioning of silver complex **3** around the water chain results in a suprastructure that also maintains the helicity of the water cluster (Figure 10).

**Figure 10:**
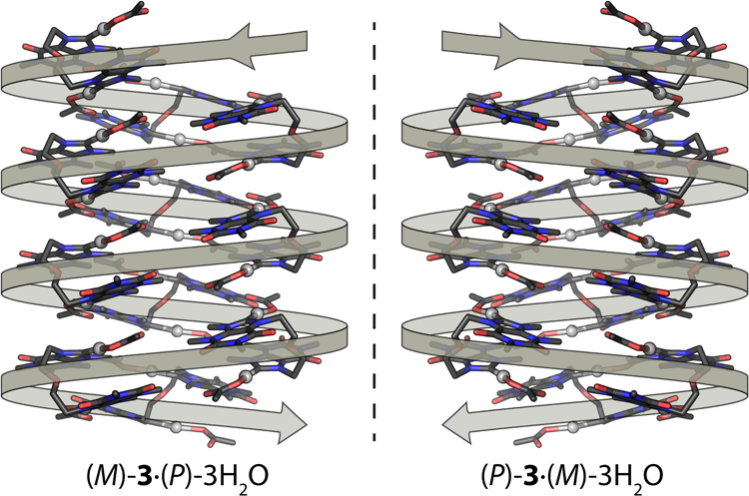
Two enantiomeric helical superstructures within the crystal packings of **3**·3H_2_O.

To summarize, the transfer of chirality by a single achiral molecule is fundamentally limited. As demonstrated here, this situation changes when achiral molecules self-organize into supramolecular chiral assemblies. Such systems can generate chirality through interactions and molecular recognition, even in the absence of intrinsic chirality in the individual components.

## Conclusions

The synthesis of the ether-nucleobase derivative **1** and the ditopic proligands **2**[OTf]_2_ and **2**[Br]_2_ facilitated the preparation of the silver-nucleobase complex **3**, which was crystallized in DMSO and in chlorinated solvents.

In crystals grown from DMSO, complex **3** adopts two enantiomeric isomers, namely *P* and *M*, which arise from rotation around the ether bridge. Within the crystal lattice, both enantiomers alternate in intermolecular π–π interactions, stabilizing a racemic ladder-like structure. When crystallized from wet apolar solvents such as CHCl_3_ or CH_2_Cl_2_, crystalline prisms of **3**·3H_2_O assemble to achieve 1D helical water columns. Moreover, (*M*)-**3**·(*P*)-3H_2_O crystallizes in the *P*3_1_21 space group, where **3** adopts *M*-chirality, while the water array exhibits *P*-helicity. In contrast, the enantiomeric crystal (*P*)-**3**·(*M*)-3H_2_O is assigned to the *P*3_2_21 space group.

The crystal lattice of **3**·3H_2_O is primarily stabilized by hydrogen bonding interactions, with other packing and intermolecular forces contributing to a lesser extent. This suggests that the arrangement of water molecules and their interactions with the silver complex **3** are fundamental to its structural integrity.

Therefore, chiral resolution of enantiomers during crystallization occurs spontaneously, without chiral auxiliaries. In this process, water drives the formation of enantiopure crystals, underscoring its pivotal role as a natural inducer of chirality. These findings offer fresh insights into stereochemical organization and highlight the unique ability of water to influence chiral outcomes in supramolecular assemblies.

## Experimental Section

### General Procedures

All the reagents used in this work were purchased from commercial sources and used as received. Glassware was dried at 140 °C before use. Unless otherwise stated, all reactions were carried out under aerobic conditions. CH_2_Cl_2_ was obtained oxygen- and waterfree from a Solvent Purification System (Innovative Technologies). Crystallization procedures were performed using undried solvents. ^1^H and ^13^C­{^1^H} NMR spectra were recorded on Bruker Avance 300 (300.13 and 75.48 MHz, respectively) and Bruker Avance 400 (400.16 and 100.61 MHz, respectively) spectrometers. Spectral assignments were achieved by combination of ^1^H–^1^H COSY, ^13^C­{^1^H}-APT and ^1^H–^13^C HSQC/HMBC. NMR chemical shifts (expressed in parts per million) are referenced to residual solvent peaks (^1^H and ^13^C). Methanol was used as internal standard in ^13^C­{^1^H} and ^1^H–^13^C HSQC/HMBC spectra carried out in D_2_O. Coupling constants, *J*, are given in hertz (Hz). High-resolution electrospray mass spectra (HRMS) were acquired using a MicroTOF-Q hybrid quadrupole time-of-flight spectrometer (Bruker Daltonics, Bremen, Germany). UV–visible spectra in solution were recorded on a JASCO V-670 UV–vis spectrophotometer.

X-ray diffraction data were collected on a Bruker D8 Venture diffractometer, using graphite-monochromated Mo Kα radiation (λ = 0.71073 Å). Diffracted intensities were integrated and corrected for absorption effects using the multiscan method.^50–52^ Both procedures are included in the APEX4 package. All the structures were solved by direct methods with SHELXS^53^ and refined by full-matrix least-squares on *F* ^2^ with SHELXL.^54^

#### Crystal Data for Compound **1**·0.25H_2_O

C_72_H_90_N_32_O_21_, *M*_r_ = 1739.75, colorless plate, monoclinic, *P*21/*c*, *a* = 7.2685(3) Å, *b* = 15.7196(6) Å, *c* = 17.4713(7) Å, β = 101.1997(13)°, *V* = 1958.22(14) Å^3^, *Z* = 1, *T* = 100(2) K, *D*_calcd_ = 1.475 g cm^–3^, μ = 0.112 mm^–1^, absorption correction factors min. 0.090 max. 0.988, 69364 reflections, 4890 unique (*R*_int_ = 0.1032), 3432 observed, *R*_1_ = 0.0491 [I > 2σ­(I)], w*R*_2_(*F* ^2^) = 0.1322 (all data), GOF = 1.018. CCDC 2410723.

#### Crystal Data for Compound **2**[OTf]_2_

C_22_H_28_F_6_N_8_O_11_S_2_, *M*_r_ = 758.64, colorless prism, triclinic *P*–1, *a* = 9.4248(8) Å, *b* = 12.1373(11) Å, *c* = 13.4277(12) Å, α = 92.6242(12)°, β = 90.4004(12)°, γ = 103.6345(12)°, *V* = 1490.9(2) Å^3^, *Z* = 2, *T* = 100(2) K, *D*_calcd_ = 1.690 g cm^–3^, μ = 0.289 mm^–1^, absorption correction factors min. 0.822 max. 0.918, 19195 reflections, 7261 unique (*R*_int_ = 0.0408), 5185 observed, *R*_1_ = 0.0484 [I > 2σ­(I)], w*R*_2_(*F* ^2^) = 0.1147 (all data), GOF = 1.041. CCDC 2410724.

#### Crystal Data for Compound **2**[Br]_2_·CH_3_OH

C_21_H_32_Br_2_N_8_O_6_, *M*_r_ = 652.36, colorless block, monoclinic *P21/c*, *a* = 8.4766(3) Å, *b* = 22.1517(6) Å, *c* = 14.5982(5) Å, β = 103.2819(13)°, *V* = 2667.80(15) Å^3^, *Z* = 4, *T* = 100(2) K, *D*_calcd_ = 1.624 g cm^–3^, μ = 3.091 mm^–1^, absorption correction factors min. 0.739 max. 0.790, 109976 reflections, 6638 unique (*R*_int_ = 0.0456), 6088 observed, *R*_1_ = 0.0333 [I > 2σ­(I)], w*R*_2_(*F* ^2^) = 0.0938 (all data), GOF = 1.069. CCDC 2410725.

#### Crystal Data for Compound **3**·2DMSO

C_28_H_44_Ag_2_N_8_O_11_S_2_, *M*_r_ = 948.57, colorless block, monoclinic *P*21/*c*, *a* = 20.1200(11) Å, *b* = 8.5680(5) Å, *c* = 22.0957(12) Å, β = 106.777(2)°, *V* = 3646.9(4) Å^3^, *Z* = 4, *T* = 100(2) K, *D*_calcd_ = 1.728 g cm^–3^, μ = 1.256 mm^–1^, absorption correction factors min. 0.727 max. 0.828, 107317 reflections, 9107 unique (*R*_int_ = 0.0511), 7984 observed, *R*_1_ = 0.0354 [I > 2σ­(I)], w*R*_2_(*F* ^2^) = 0.0899 (all data), GOF = 1.158. CCDC 2410726.

#### Crystal Data for Compound (*M*)-**3**·(*P*)-3H_2_O

C_24_H_38_Ag_2_N_8_O_12_, *M*_r_ = 846.36, colorless prism, trigonal *P*3121, *a* = 19.5911(6) Å, *c* = 7.0016(3) Å, *V* = 2327.26(17)­Å^3^, *Z* = 3, *T* = 100(2) K, *D*_calcd_ = 1.812 g cm^–3^, μ = 1.336 mm^–1^, absorption correction factors min. 0.842 max. 0.899, 75560 reflections, 3880 unique (*R*_int_ = 0.0545), 3763 observed, *R*_1_ = 0.0938 [I > 2σ­(I)], w*R*_2_(*F* ^2^) = 0.1966 (all data), GOF = 1.092. CCDC 2410727.

#### Crystal Data for Compound (*M*)-**3**·(*P*)-3H_2_O

C_24_H_38_Ag_2_N_8_O_12_, *M*_r_ = 846.36, colorless prism, trigonal *P*3221, *a* = 19.5955(4) Å, *c* = 7.0045(3) Å, *V* = 2329.27(14) Å^3^, *Z* = 3, *T* = 100(2) K, *D*_calcd_ = 1.810 g cm^–3^, μ = 1.335 mm^–1^, absorption correction factors min. 0.805 max. 0.923, 90779 reflections, 3878 unique (*R*_int_ = 0.0553), 3729 observed, *R*_1_ = 0.0902 [I > 2σ­(I)], w*R*_2_(*F* ^2^) = 0.2029 (all data), GOF = 1.097. CCDC 2410728.

### Synthesis of Bis­(theophylline-*N*7-ethyl)­ether, **1**

Theophylline (3.30 g, 18.3 mmol) and NaOH (1.06 g, 26.5 mmol) were suspended in 20 mL of DMF and the mixture was heated to 50 °C. Then, bis­(2-chloroethyl) ether (1.3 mL, 11.1 mmol) was added and the temperature was raised to 80 °C. After 40 h the mixture was cooled down to room temperature and 20 mL of water were added. The white suspension was filtered, washed with water (2 × 10 mL) and methanol (2 × 5 mL) and dried under vacuum to yield **1** as a white solid (3.00 g, 76%). ^1^H NMR (400 MHz, CDCl_3_, 298 K): δ 7.40 (s, 2H, H8), 4.37 (t, ^3^ *J*_H–H_ = 4.5 Hz, 4H, *N*7-CH_2_), 3.71 (t, ^3^ *J*_H–H_ = 4.5 Hz, 4H, *O*-CH_2_), 3.55 (s, 6H, *N*3-CH_3_), 3.34 (s, 6H, *N*1-CH_3_). ^13^C­{^1^H} NMR (100 MHz, CDCl_3_, 298 K): δ 155.30 (C6), 151.63 (C2), 149.08 (C4), 141.93 (C8), 106.56 (C5), 69.38 (*O*-C), 46.89 (*N*7-C), 29.88 (*N*3-C), 28.02 (*N*1-C). HRMS (CHCl_3_/CH_3_CN) [M + Na]^+^ calcd. for C_18_H_22_N_8_O_5_Na: 453.1605; found: 453.1603.

### Synthesis of Bis­(theophylline-*N*9-methyl-*N*7-ethyl)­ether Triflate, **2**[OTf]_2_

Under argon atmosphere, methyl trifluoromethanesulfonate (0.75 mL, 6.65 mmol) was added to a suspension of **1** (1.00 g, 2.34 mmol) in 20 mL of dry dichloromethane and the mixture was stirred at room temperature. The suspension gradually disappeared upon 1 h of stirring, giving rise to a colorless solution. Shortly after, a white precipitate appeared, and the mixture was stirred at room temperature for 3 days. Filtration and washings with dichloromethane (2 × 10 mL) and diethyl ether (3 × 10 mL) afforded **2**[OTf]_2_ as a white solid (1.56 g, 2.06 mmol, 88%). ^1^H NMR (400 MHz, CD_3_CN, 298 K): δ 8.79 (s, 2H, H8), 4.67 (t, ^3^ *J*_H–H_ = 4.8 Hz, 4H, *N*7-CH_2_), 4.14 (s, 6H, *N*9-CH_3_), 3.81 (t, ^3^ *J*_H–H_ = 4.8 Hz, 4H, *O*-CH_2_), 3.74 (s, 6H, *N*3-CH_3_), 3.31 (s, 6H, *N*1-CH_3_). ^13^C­{^1^H} NMR (100 MHz, CD_3_CN, 298 K): δ 154.75 (C6), 151.45 (C2), 140.83 (C4), 140.11 (C8), 108.80 (C5), 68.63 (*O*-C), 49.83 (*N*7-C), 38.09 (*N*9-C), 32.22 (*N*3-C), 29.14 (*N*1-C). ^19^F NMR (282 MHz, CD_3_CN, 298 K): δ −79.40 (s, OTf). HRMS (CH_3_CN) [M – 2OTf]^2+^ calcd. for C_20_H_28_N_8_O_5_: 230.1086; found: 230.1093/[M – OTf]^+^ calcd. for C_21_H_28_N_8_O_8_SF_3_: 609.1697; found: 609.1727.

### Synthesis of Bis­(theophylline-*N*9-methyl-*N*7-ethyl)­ether Bromide, **2**[Br]_2_

**2**[OTf]_2_ (298 mg, 0.39 mmol) was suspended in 15 mL of THF and tetrabutylammonium bromide (326 mg, 1.01 mmol) was added. After stirring at room temperature for 15 h, the white precipitate was filtered off, washed with further THF and acetone (2 × 5 mL) and dried under vacuum to yield **2**[Br]_2_ as a white solid (240 mg, 0.38 mmol, 98%). ^1^H NMR (300 MHz, D_2_O, 298 K): δ 4.75 (t, ^3^ *J*_H–H_ = 4.9 Hz, 4H, *N*7-CH_2_), 4.24 (s, 6H, *N*9-CH_3_), 3.96 (t, ^3^ *J*_H–H_ = 4.9 Hz, 4H, *O*-CH_2_), 3.83 (s, 6H, *N*3-CH_3_), 3.37 (s, 6H, *N*1-CH_3_). ^13^C­{^1^H} NMR (100 MHz, D_2_O, 298 K): δ 155.12 (C6), 152.09 (C2), 140.46 (C4), 140.12 (t, ^1^ *J*_C–D_ = 30.0 Hz, C8), 108.84 (C5), 68.64 (*O*-C), 49.25 (*N*7-C), 37.93 (*N*9-C), 32.38 (*N*3-C), 29.31 (*N*1-C). HRMS (CH_3_OH) [M – 2Br]^2+^ calcd. for C_20_H_28_N_8_O_5_: 230.1086; found: 230.1093/[M – Br]^+^ calcd. for C_20_H_28_N_8_O_5_Br: 541.1343; found: 541.1349.

### Synthesis of **3**

**2**[Br]_2_ (111 mg, 0.18 mmol) was suspended in 12 mL of dichloromethane and silver acetate (129 mg, 0.77 mmol) was added. The white precipitate gradually transformed into a yellowish suspension in the span of 15 min. After stirring overnight, the yellow solid was removed upon filtration through Celite and the colorless solution was concentrated to about 1 mL under reduced pressure. Addition of 10 mL of hexane afforded a white solid which was filtered, washed with further hexane (2 × 5 mL) and dried under vacuum (110 mg, 0.14 mmol, 77%). ^1^H NMR (400 MHz, CD_2_Cl_2_, 298 K): 4.59 (pt, ^3^ *J*_H–H_ = 4.2 Hz, 4H, *N*7-CH_2_), 4.27 (s, 6H, *N*9-CH_3_), 3.86 (br-pt, 4H, *O*-CH_2_), 3.84 (s, 6H, *N*3-CH_3_), 3.35 (s, 6H, *N*1-CH_3_), 1.90 (s, 6H, *OAc*-CH_3_). ^13^C­{^1^H} NMR (100 MHz, CD_2_Cl_2_, 298 K): 187.06 (C8), 177.57 (C13), 154.03 (C6), 151.32 (C2), 141.41 (C4), 109.46 (C5), 69.27 (*O*-C), 52.19 (*N*7-C), 40.47 (*N*9-C), 32.32 (*N*3-C), 28.99 (*N*1-C), 22.56 (C14). HRMS (CDCl_3_/CH_3_OH) [M – Ag – 2 OAc]^+^ calcd. for C_20_H_26_N_8_O_5_Ag: 565.1072; found: 565.1049.

## Supplementary Material



## Notes

1D and 2D NMR spectra for all compounds (PDF)
