# Supplementary material for: Water-Mediated Chiral Resolution of Ag–NHC(Nucleobase) Complexes
Source: Inorg Chem. 2025 Feb 10;64(11):5487–94. doi: 10.1021/acs.inorgchem.4c05384 (PMC12124713; doi:10.1021/acs.inorgchem.4c05384)
Supplement: Supplementary file 1 [file ic4c05384_si_001.pdf]

Supporting information

Water-Mediated Chiral Resolution of Ag–NHC(Nucleobase) Complexes

Alvaro Polo, Ricardo Rodríguez, Ramón Macías, Daniel Cobo Paz and Pablo J. Sanz Miguel*

Departamento de Química Inorgánica, Instituto de Síntesis Química y Catálisis Homogénea (ISQCH), Universidad de Zaragoza-CSIC, 50009, Zaragoza, Spain.

* E-mail (P. J. Sanz Miguel): pablo.sanz@unizar.es

Contents

NMR data of compounds **1**–**3** 2

UV-VIS spectra 14

X-Ray data 15

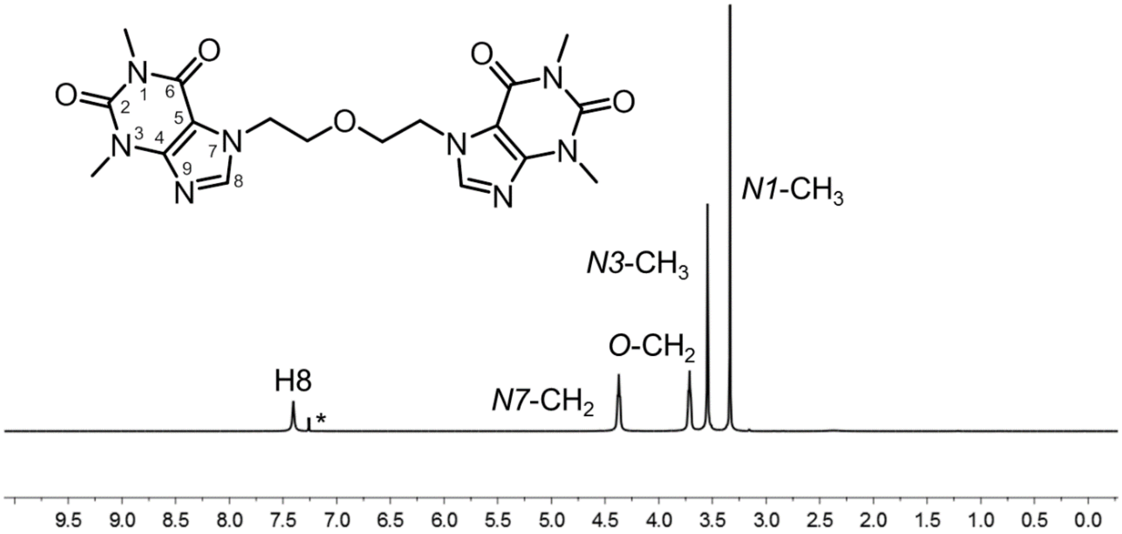

Figure S1. ^{1}H NMR spectrum of **1** (400 MHz, $CDCl_3$, 298 K).

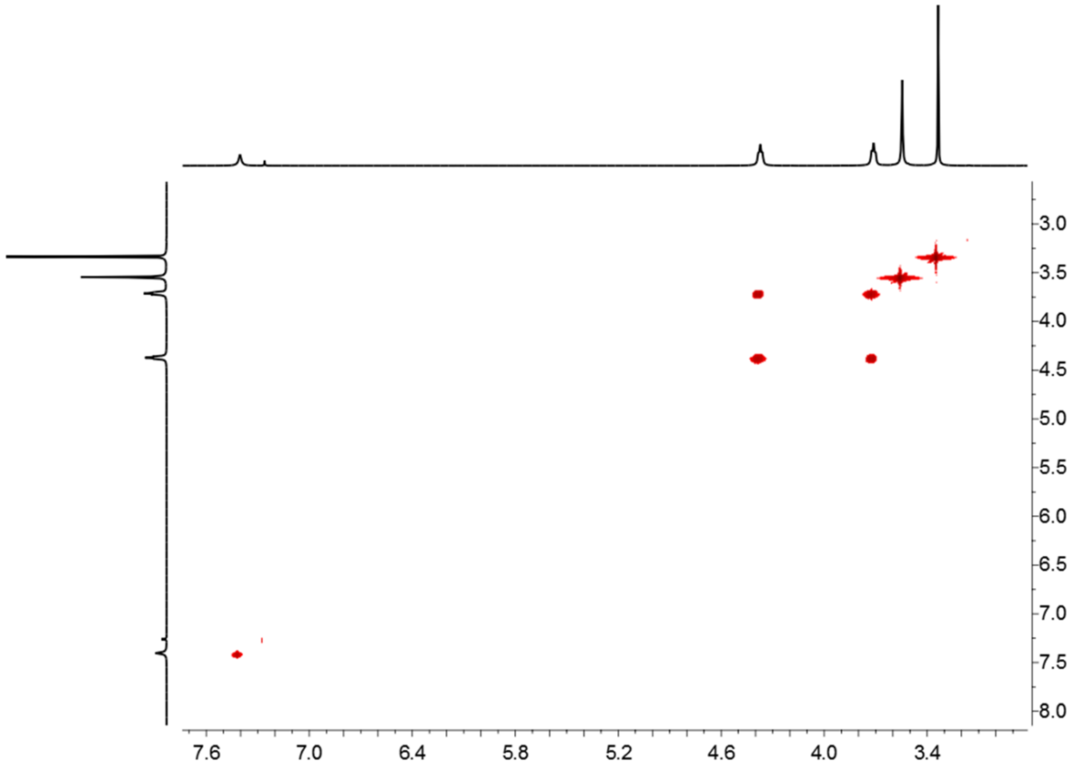

Figure S2. ^{1}H-^{1}H COSY NMR spectrum of **1** ($CDCl_3$, 298 K).

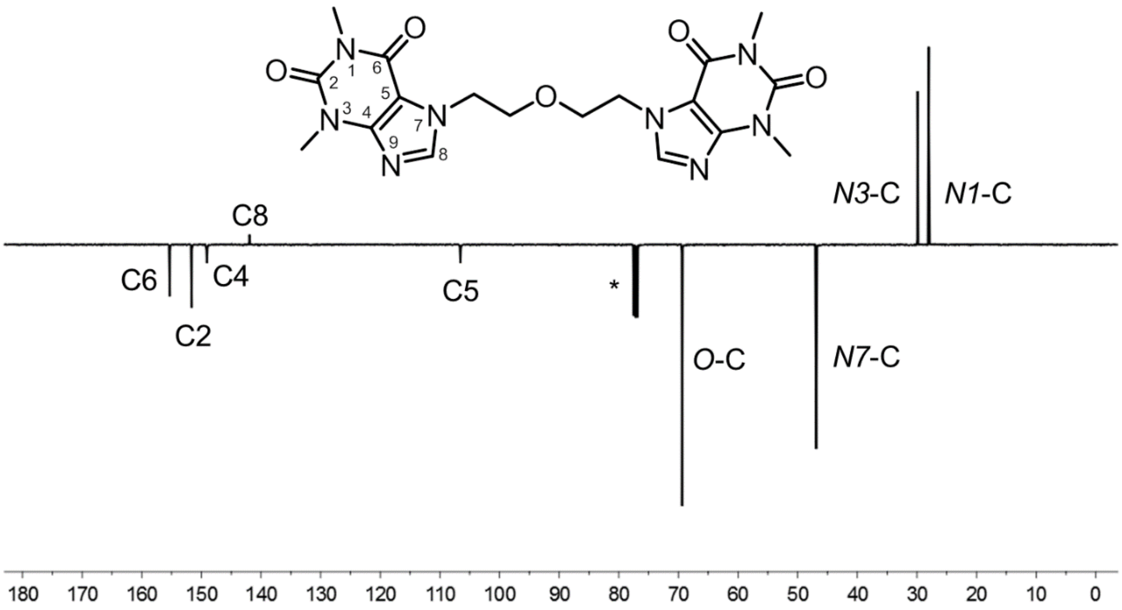

Figure S3. $^{13}C\{^{1}H\}$-APT NMR spectrum of **1** (100 MHz, $CDCl_3$, 298 K).

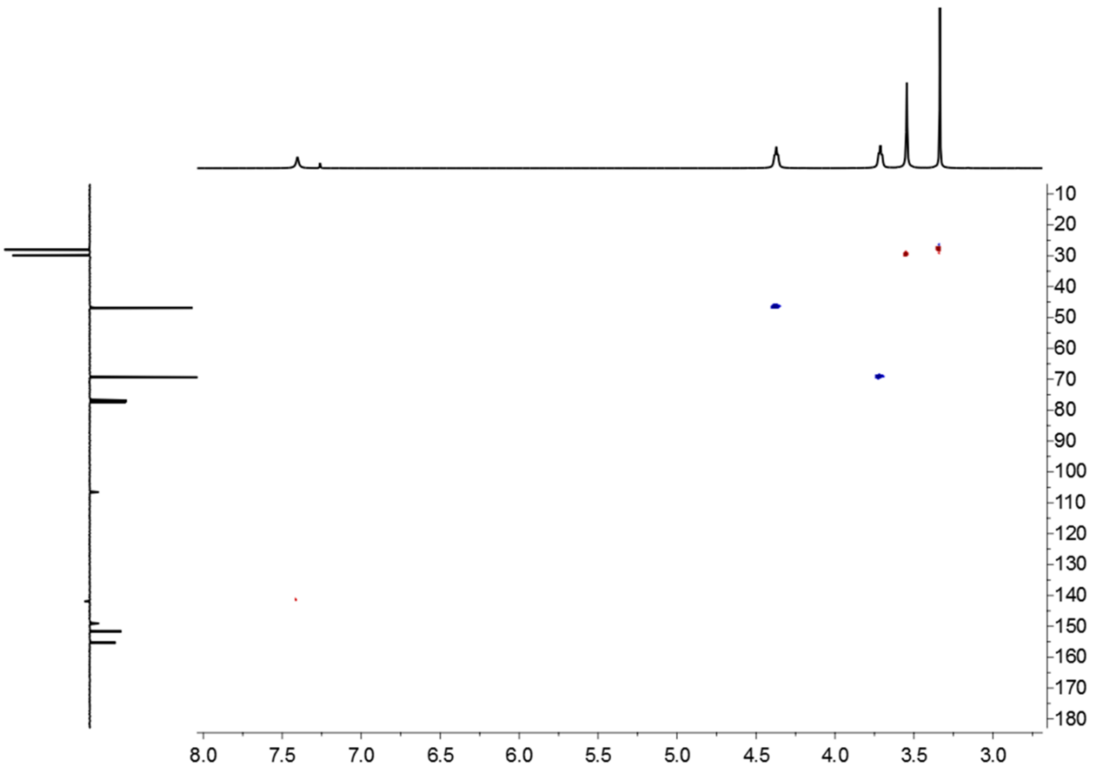

Figure S4. ^{1}H-^{13}C HSQC NMR spectrum of **1** ($CDCl_3$, 298 K).

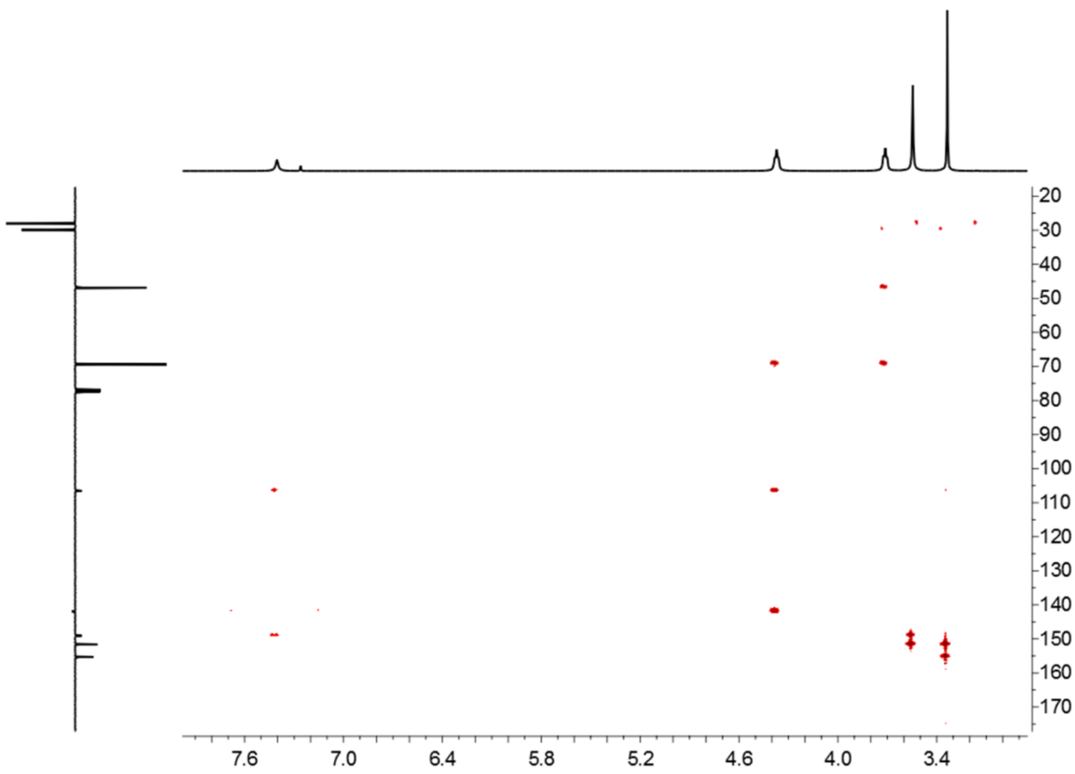

Figure S5. ^{1}H-^{13}C HMBC NMR spectrum of **1** ($CDCl_3$, 298 K).

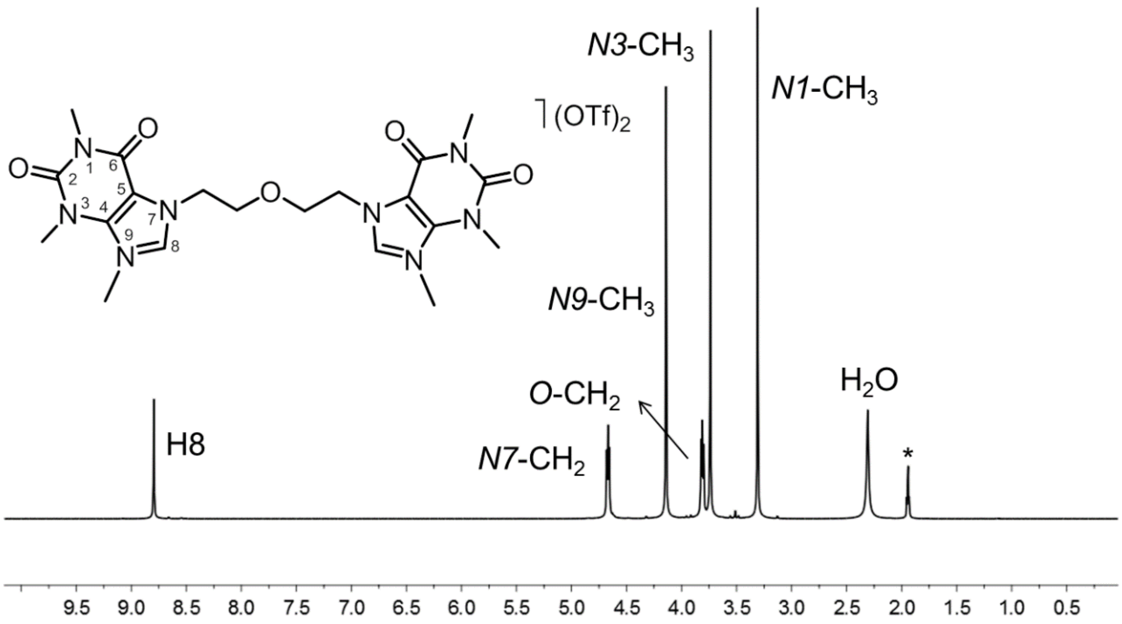

Figure S6. ^{1}H NMR spectrum of **2**[OTf]$_2$ (400 MHz, CD_3CN, 298 K).

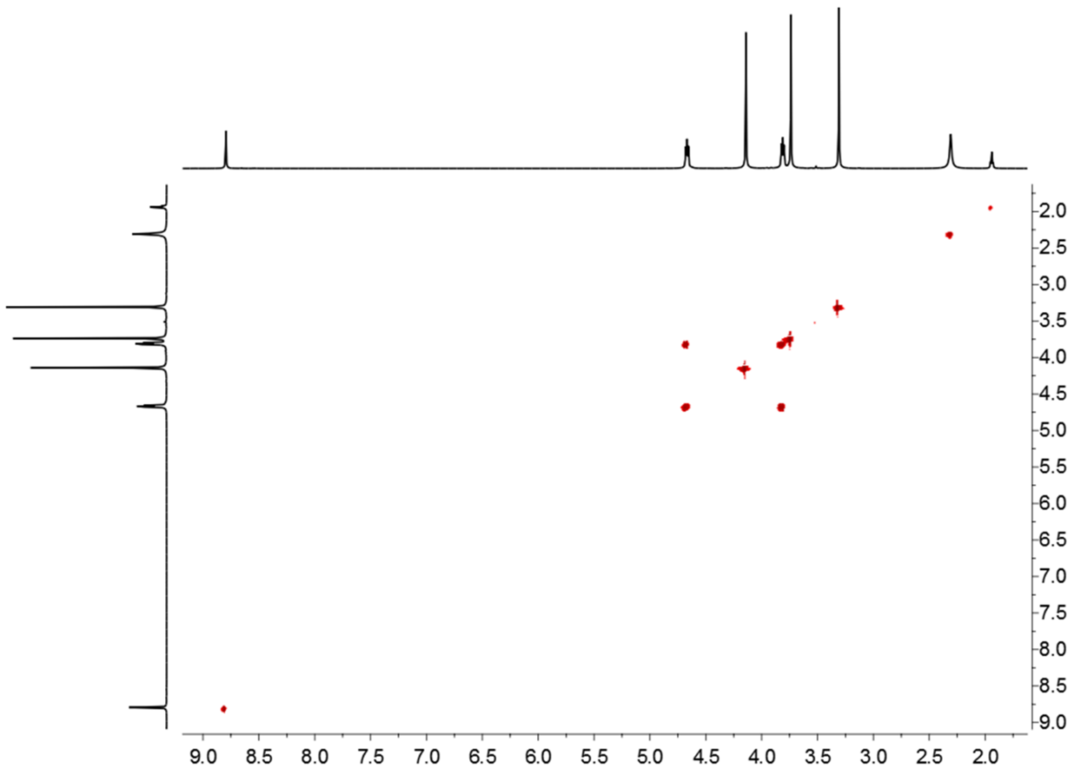

Figure S7. ^{1}H-^{1}H COSY NMR spectrum of **2**[OTf]$_2$ (CD_3CN, 298 K).

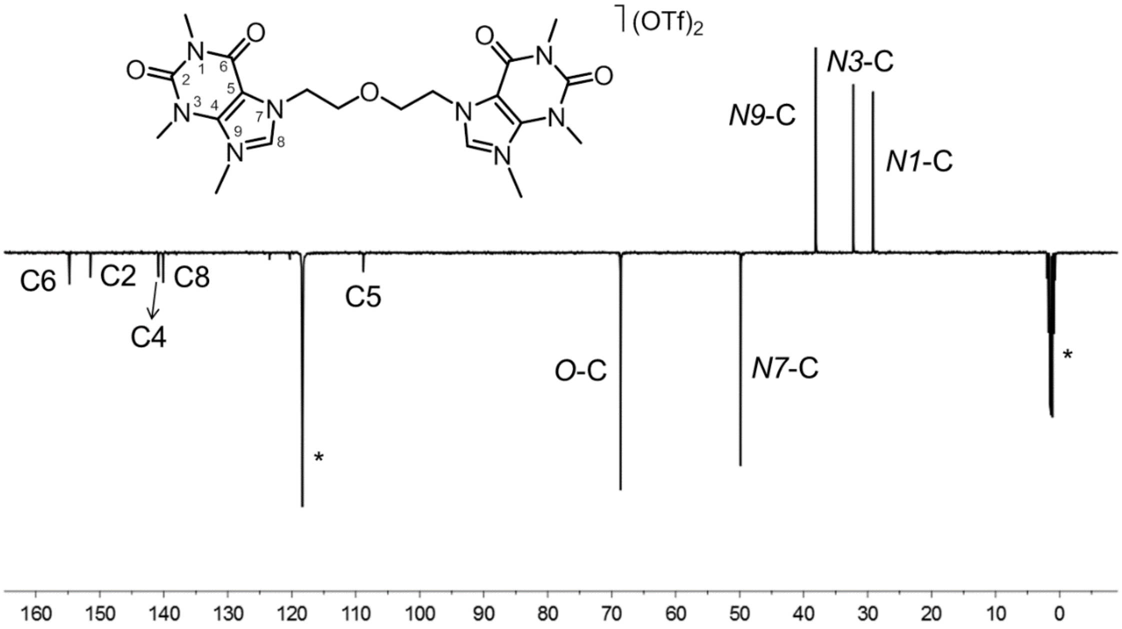

Figure S8. $^{13}C\{^{1}H\}$-APT NMR spectrum of **2**[OTf]$_2$ (100 MHz, CD_3CN, 298 K).

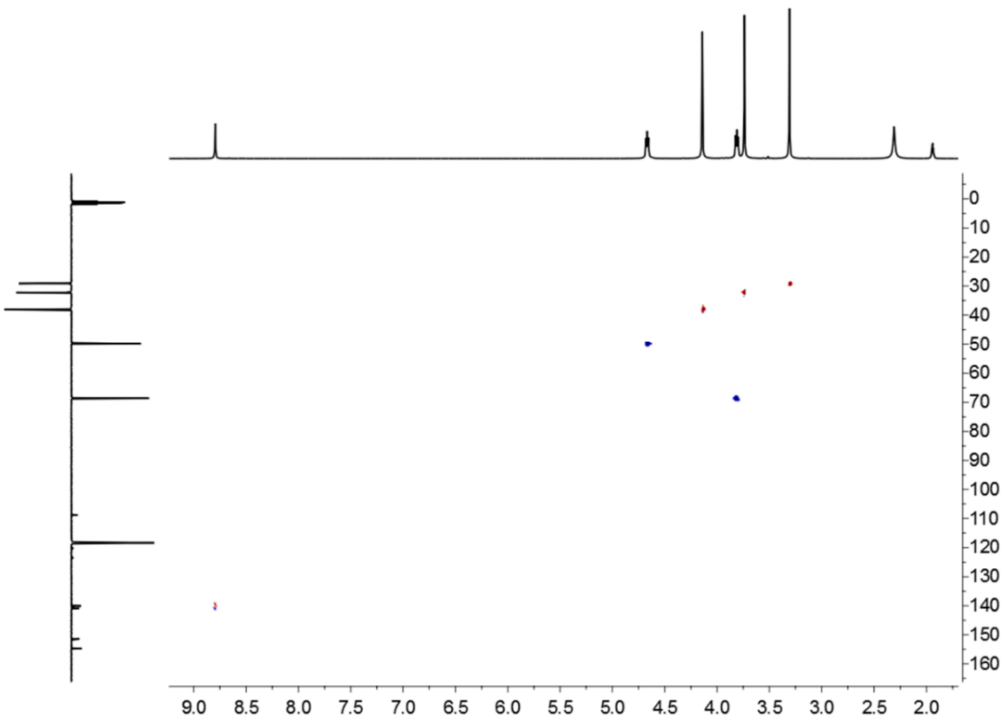

Figure S9. ^{1}H-^{13}C HSQC NMR spectrum of **2**[OTf]$_2$ (CD_3CN, 298 K).

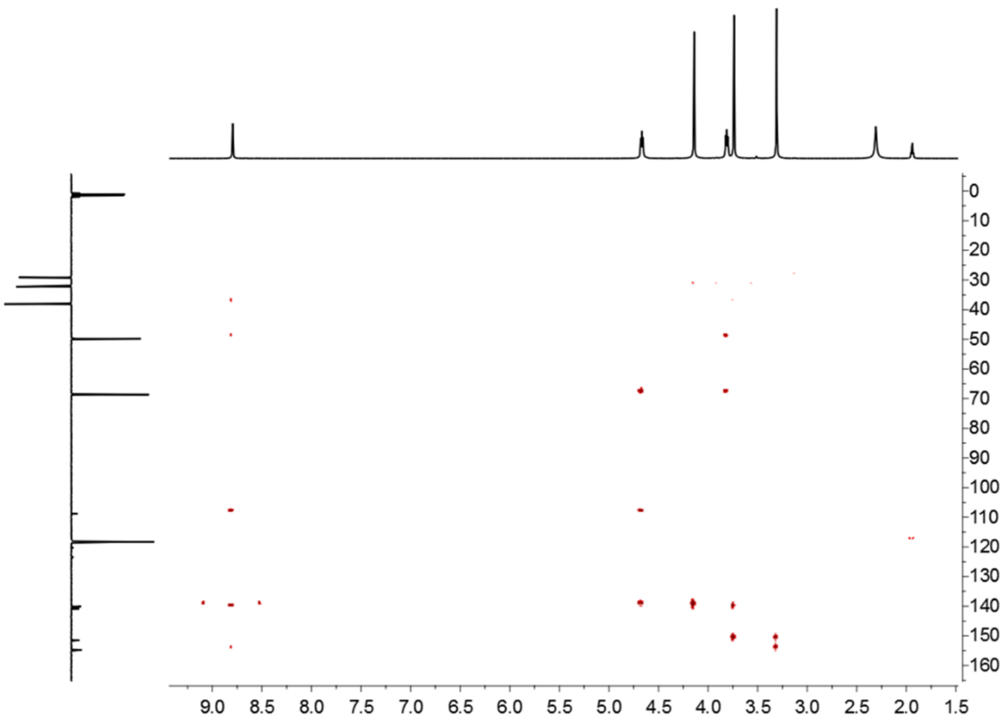

Figure S10. ^{1}H-^{13}C HMBC NMR spectrum of **2**[OTf]$_2$ (CD_3CN, 298 K).

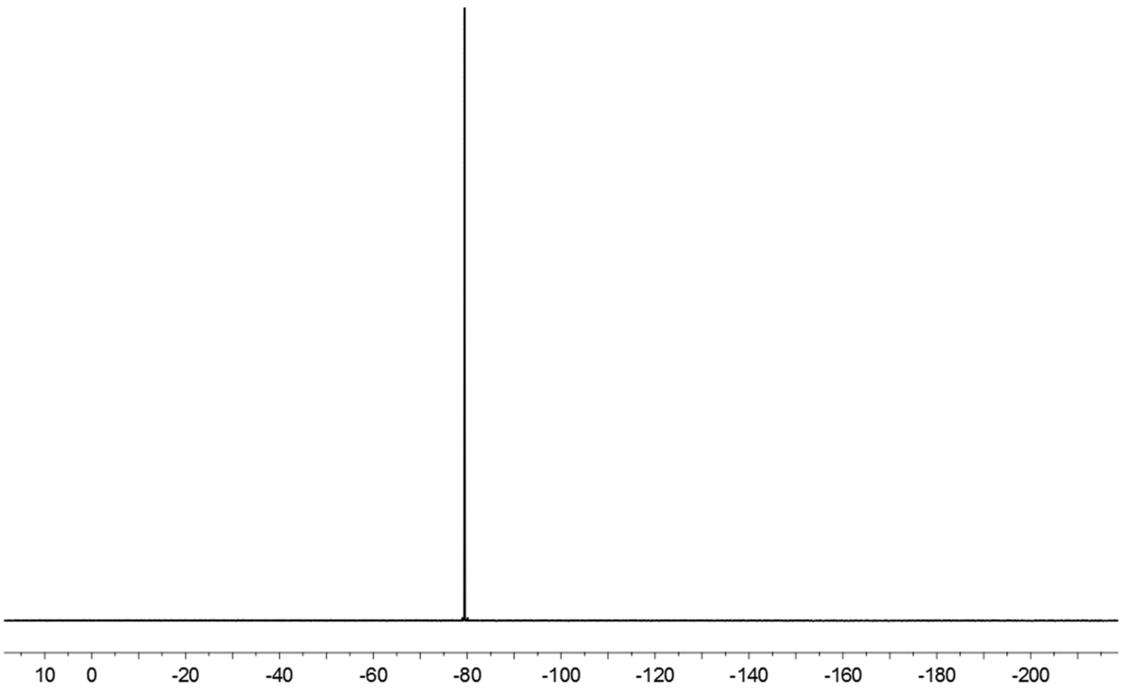

Figure S11. ^{19}F NMR spectrum of **2**[OTf]$_2$ (284 MHz, CD_3CN, 298 K).

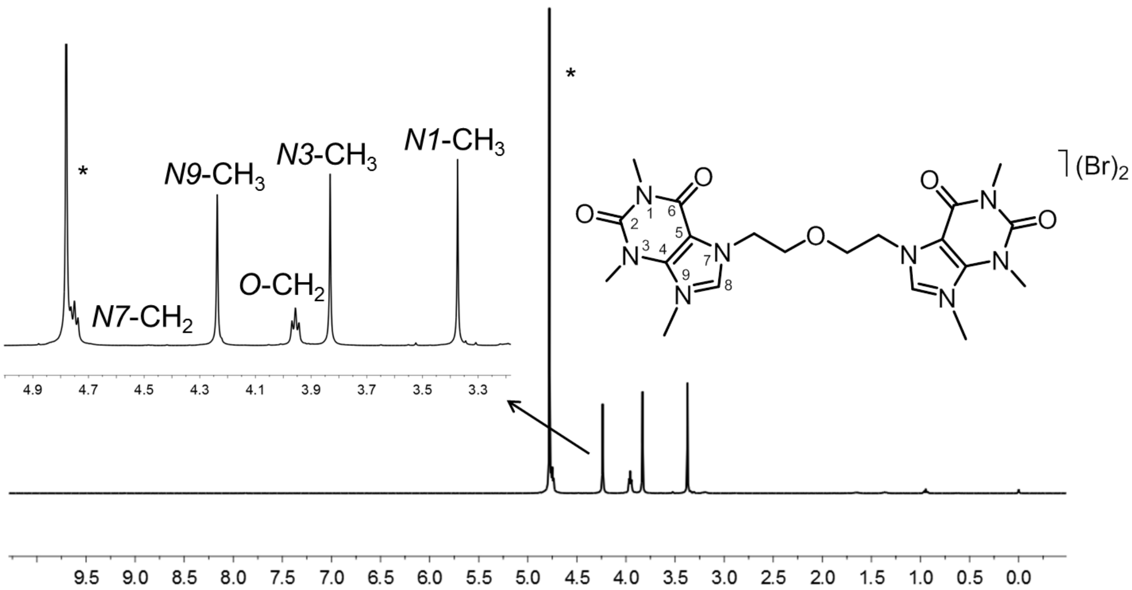

Figure S12. ^{1}H NMR spectrum of **2**[Br]$_2$ (300 MHz, D_2O, 298 K).

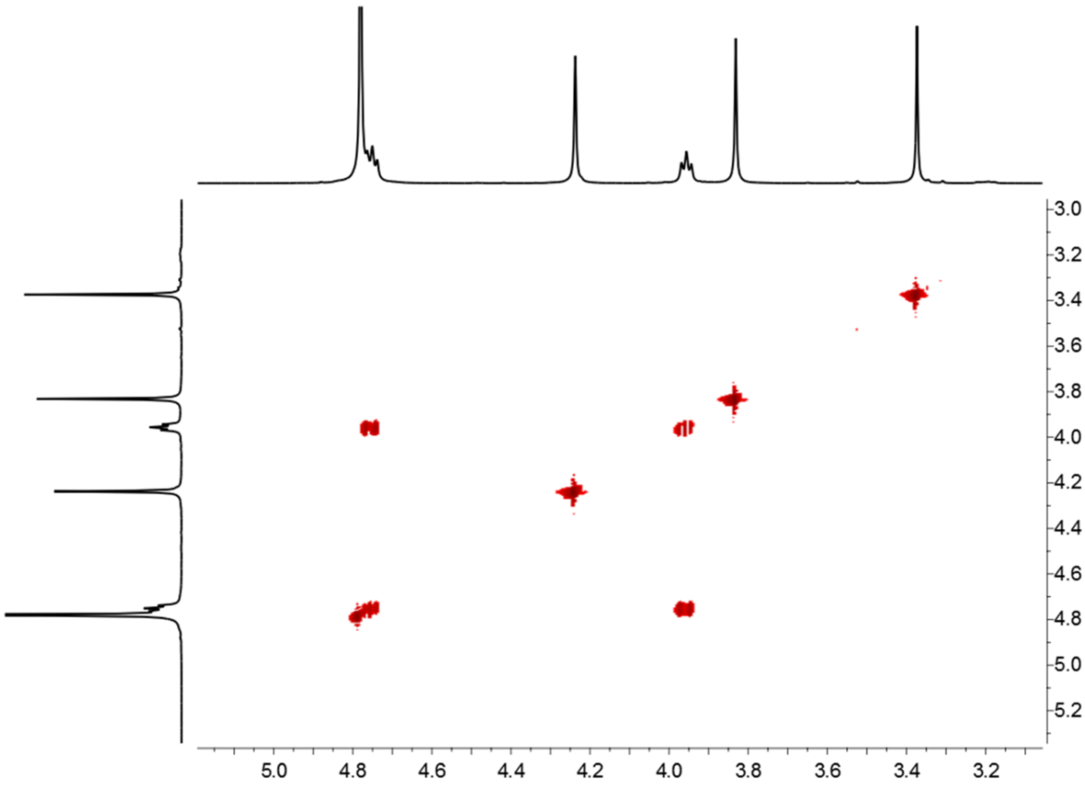

Figure S13. ^1H-^1H COSY NMR spectrum of **2**[Br]$_2$ (D_2O, 298 K).

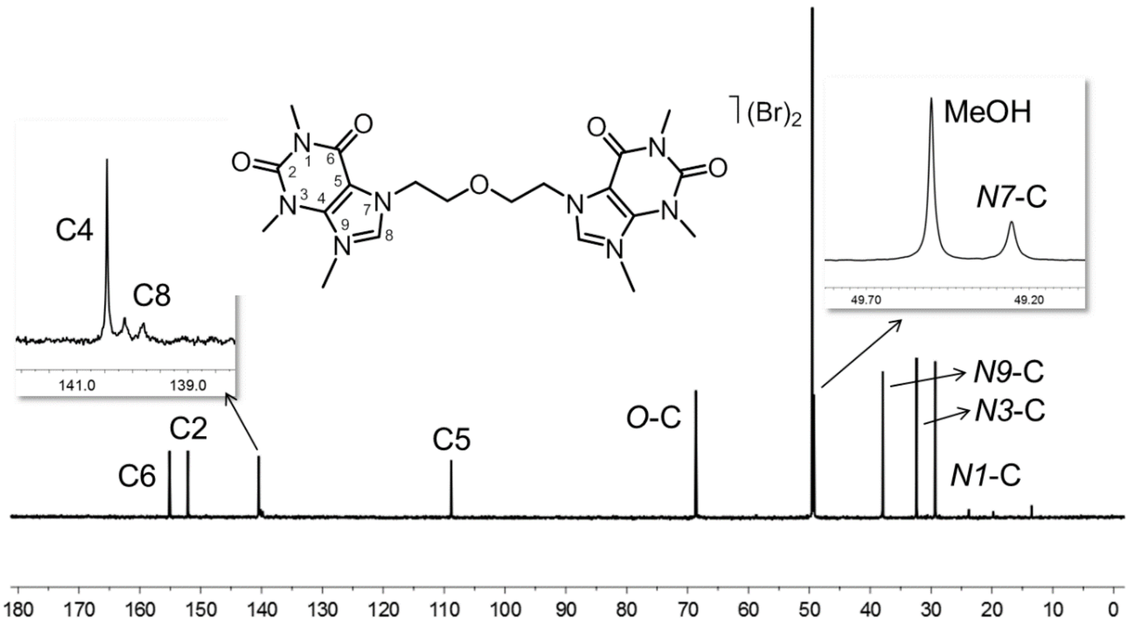

Figure S14. $^{13}C\{^1H\}$ NMR spectrum of **2**[Br]$_2$ (100 MHz, D_2O, 298 K).

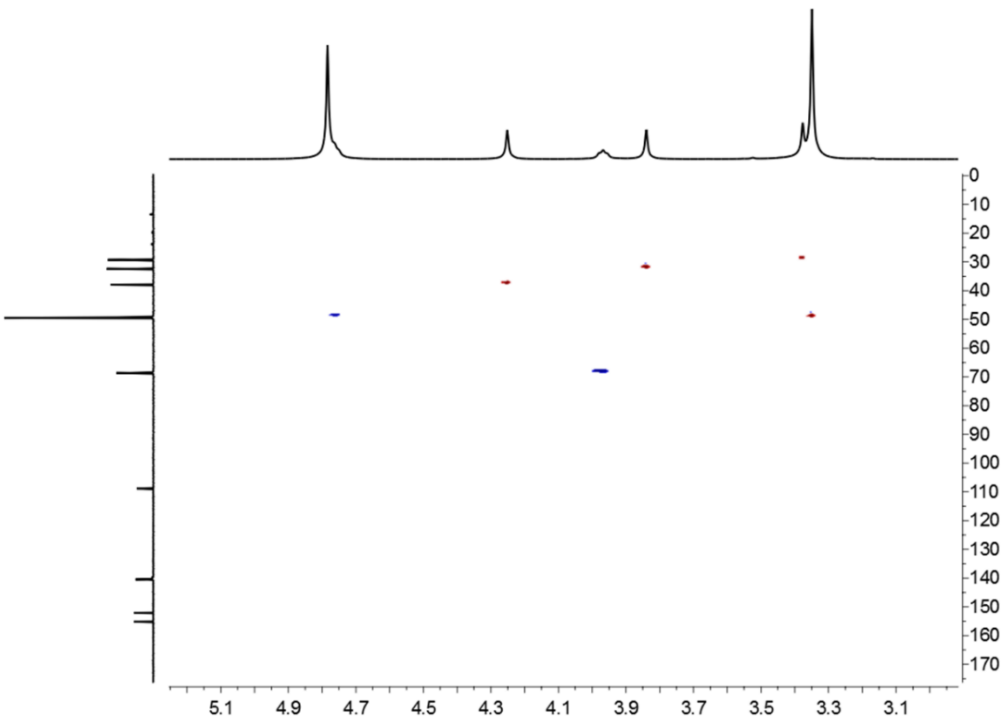

Figure S15. ^{1}H-^{13}C HSQC NMR spectrum of **2**$[Br]_2$ (D_2O, 298 K).

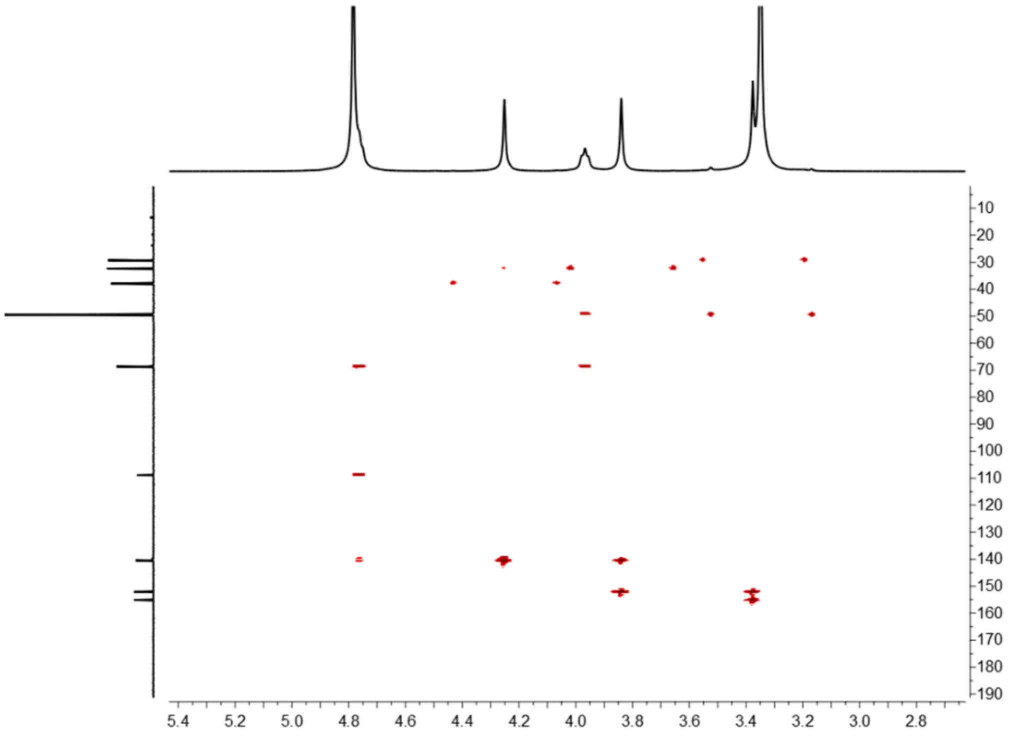

Figure S16. ^{1}H-^{13}C HMBC NMR spectrum of **2**$[Br]_2$ (D_2O, 298 K).

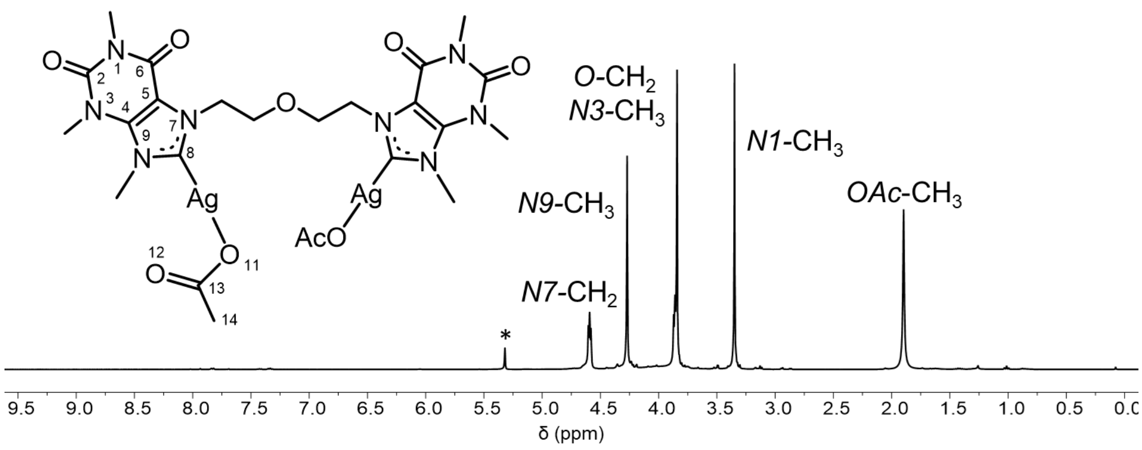

Figure S17. ^{1}H NMR spectrum of **3** (400 MHz, CD_2Cl_2, 298 K).

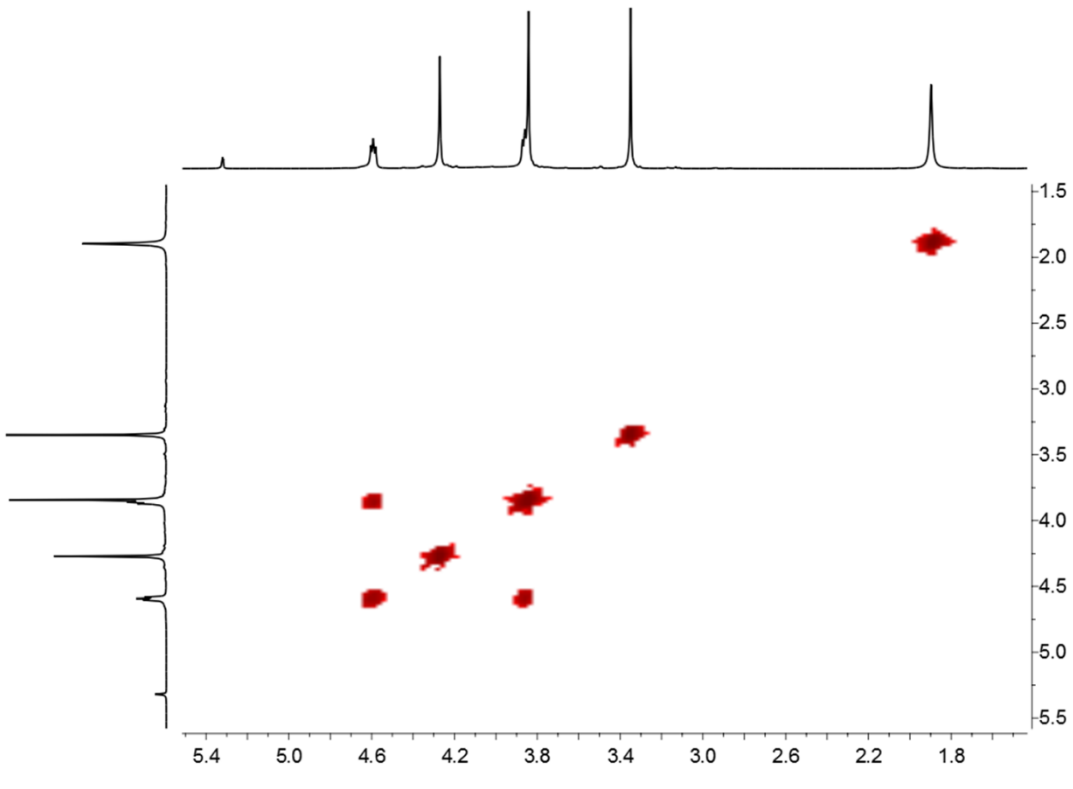

Figure S18. ^{1}H-^{1}H COSY NMR spectrum of **3** (CD_2Cl_2, 298 K).

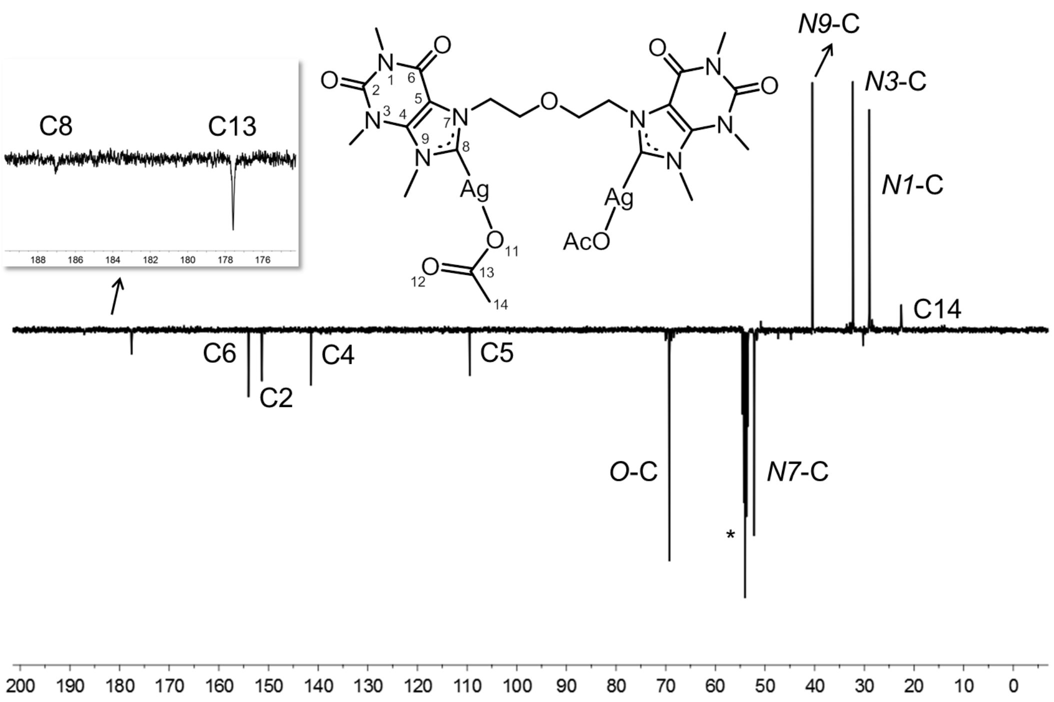

Figure S19. $^{13}C\{^{1}H\}$-APT NMR spectrum of **3** (100 MHz, CD_2Cl_2, 298 K).

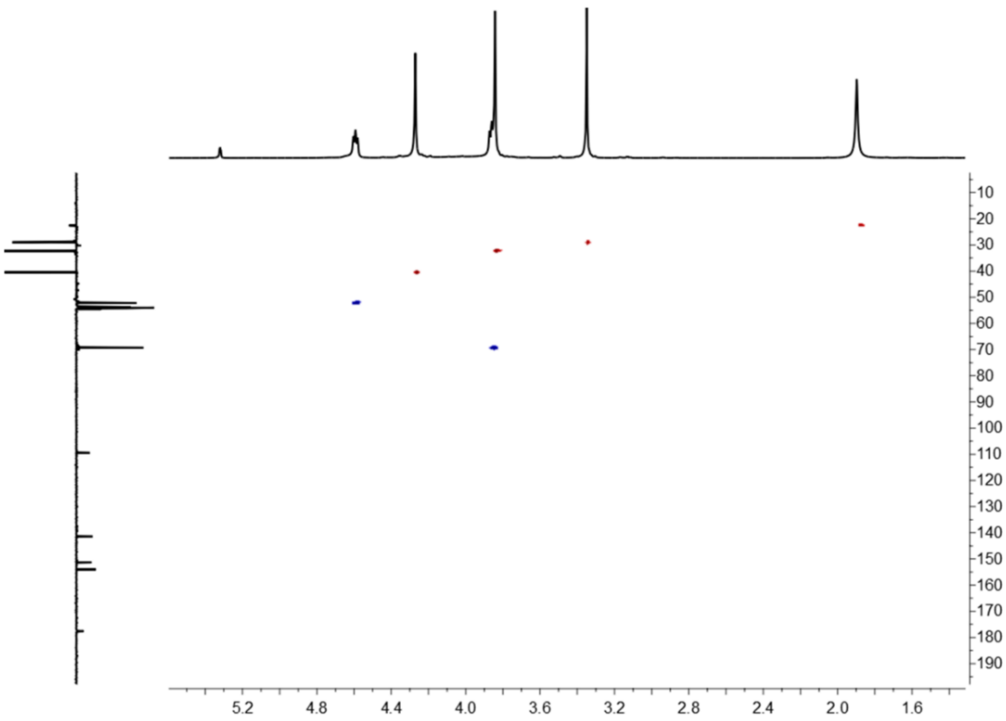

Figure S20. ^{1}H-^{13}C HSQC NMR spectrum of **3** (CD_2Cl_2, 298 K).

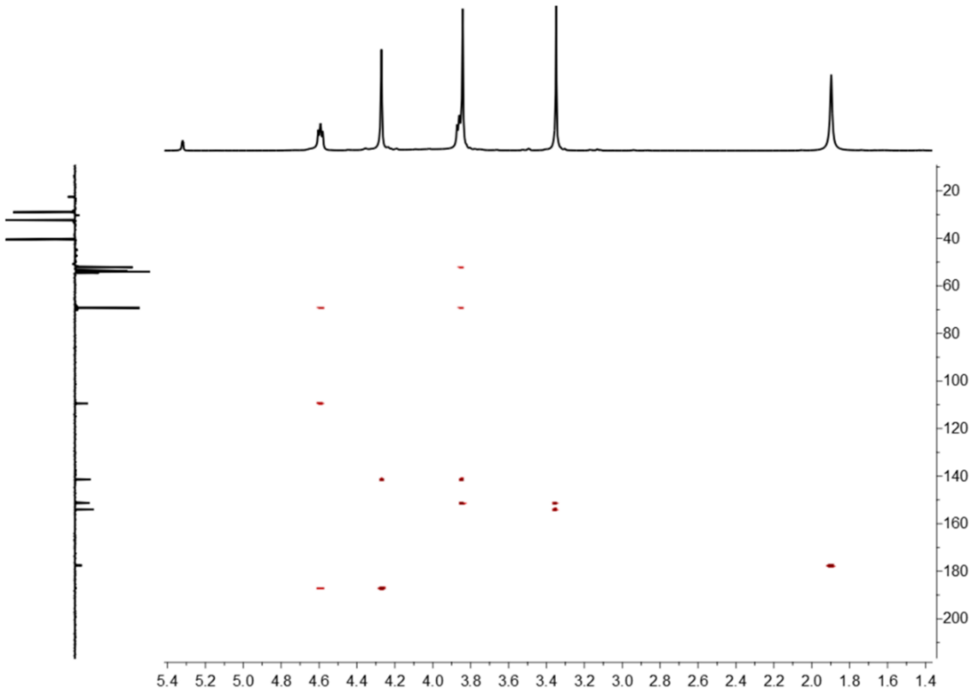

Figure S21. ^{1}H-^{13}C HMBC NMR spectrum of **3** (CD_2Cl_2, 298 K).

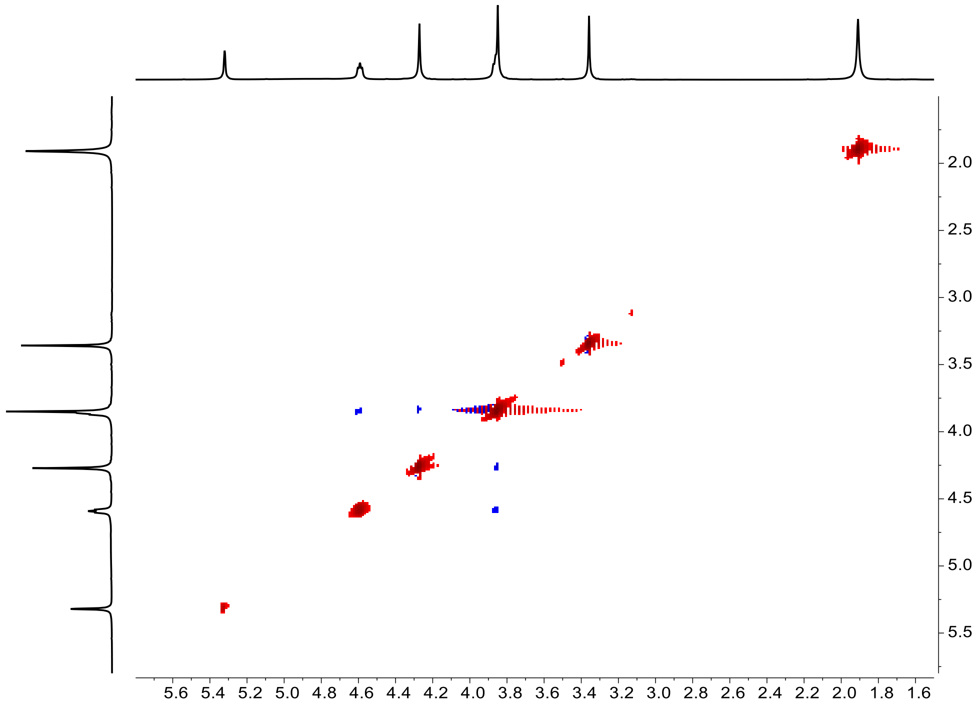

Figure S22. ^{1}H-^{1}H NOESY NMR spectrum of **3** (CD_2Cl_2, 298 K).

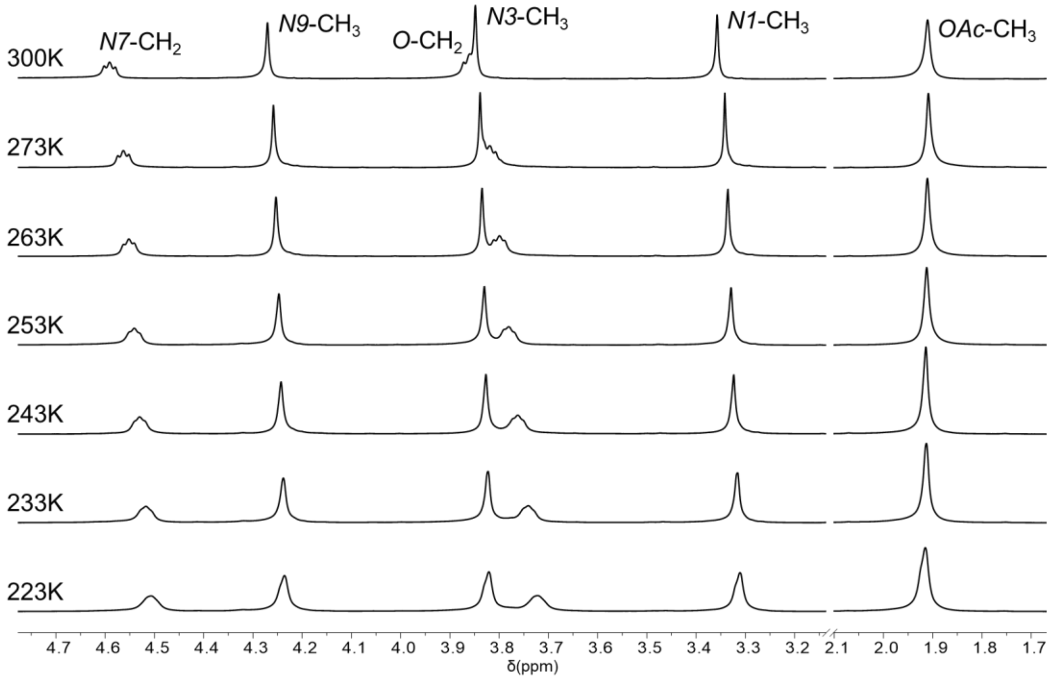

Figure S23. VT ^{1}H NMR spectrum of **3** (400 MHz, CD_2Cl_2).

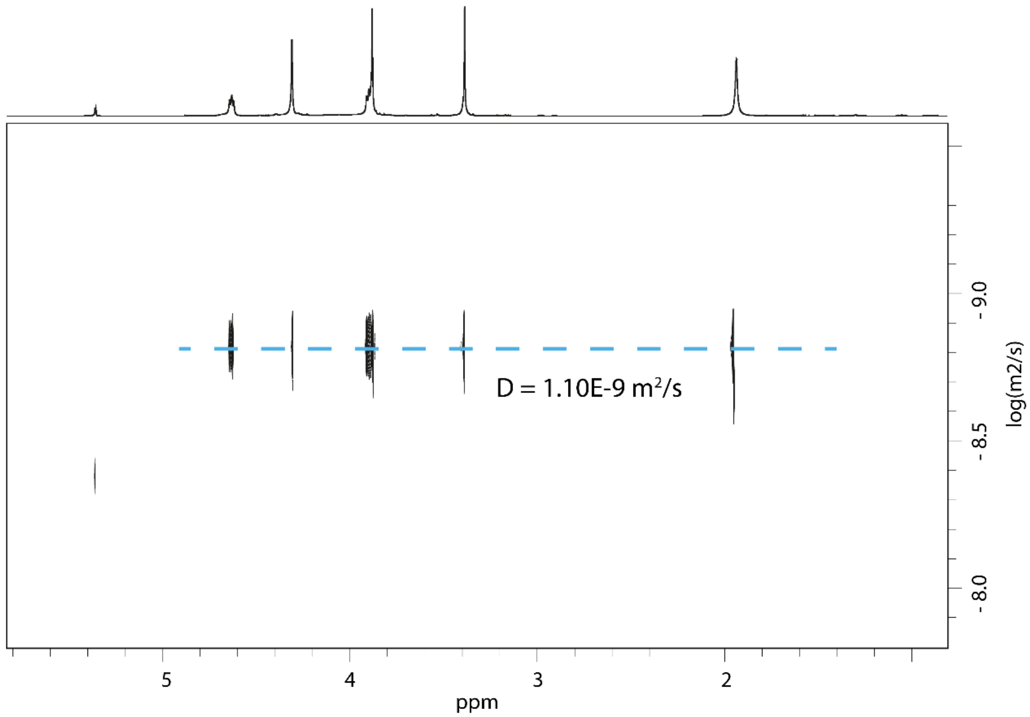

Figure S24. ^{1}H DOSY NMR spectrum of **3** (400 MHz, CD_2Cl_2, 298 K). Values for diffusion coefficients are showcased.

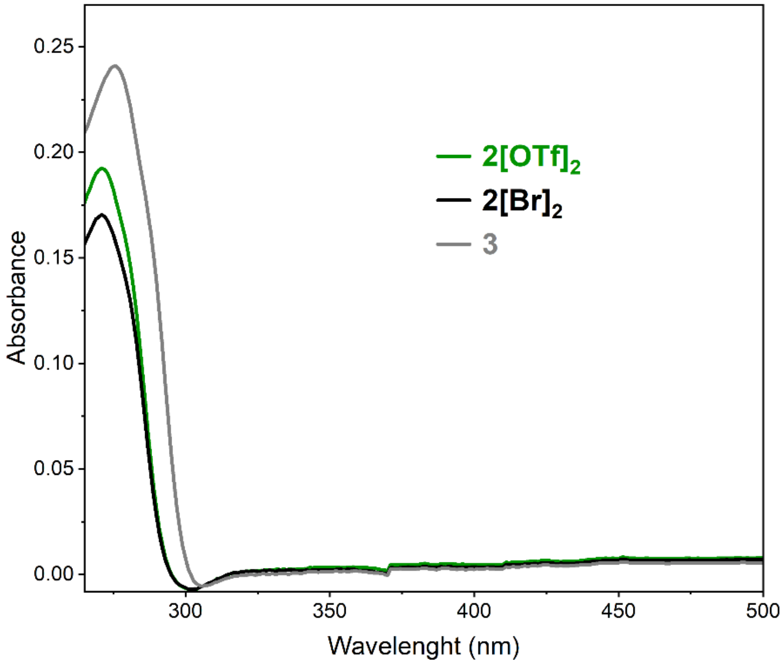

Figure S25. UV-Vis absorption spectra of **2**[OTf]$_2$, **2**[Br]$_2$ and **3** in DMSO (10^{-5} M).

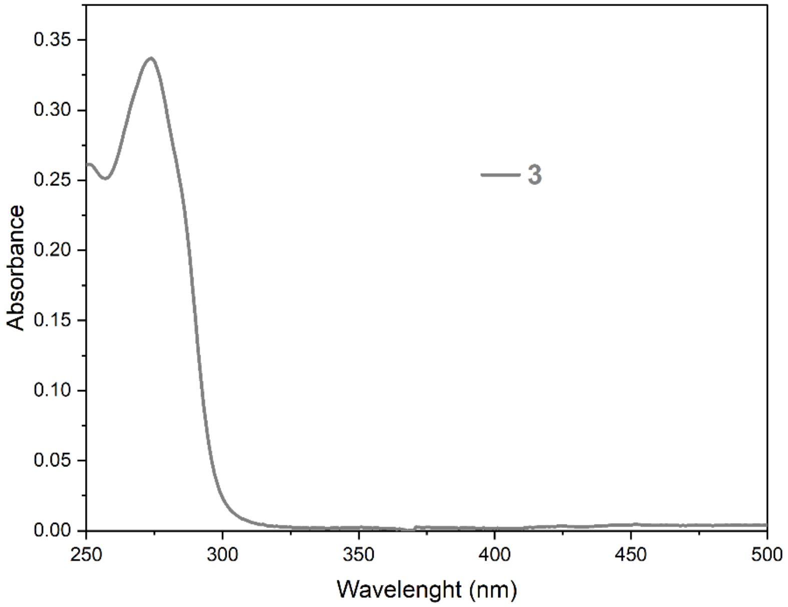

Figure S26. UV-Vis absorption spectrum of **3** in $CHCl_3$ (10^{-5} M).

Table S1. Bond distances [Å] and angles [°] in compounds **1**, **2**[OTf]$_2$, and **2**[Br]$_2$·CH_3OH.

	**1**		**2**[OTf]$_2$		**2**[Br]$_2$·CH_3OH	
	ring a	ring b	ring a	ring b	ring a	ring b
N1–C2	1.400(2)	1.401(2)	1.398(3)	1.394(3)	1.392(3)	1.398(3)
N1–C6	1.404(2)	1.408(2)	1.398(3)	1.406(3)	1.394(3)	1.403(3)
C2–N3	1.378(2)	1.380(2)	1.400(3)	1.396(3)	1.386(3)	1.387(3)
N3–C4	1.372(2)	1.377(2)	1.364(3)	1.365(3)	1.362(3)	1.364(3)
C4–C5	1.373(2)	1.371(2)	1.359(3)	1.357(3)	1.364(3)	1.366(3)
C4–N9	1.360(2)	1.357(2)	1.379(3)	1.376(3)	1.375(3)	1.374(3)
C5–C6	1.419(2)	1.419(2)	1.439(3)	1.438(3)	1.434(3)	1.435(3)
C5–N7	1.389(2)	1.385(2)	1.386(3)	1.386(3)	1.384(3)	1.383(2)
N7–C8	1.346(2)	1.348(2)	1.322(3)	1.322(3)	1.313(3)	1.320(3)
C8–N9	1.334(2)	1.338(2)	1.348(3)	1.345(3)	1.351(3)	1.352(3)
C2–N1–C6	126.45(15)	126.40(15)	126.59(19)	127.26(19)	126.20(18)	126.69(18)
N1–C2–N3	117.39(15)	117.00(15)	117.23(19)	117.1(2)	117.81(18)	117.79(18)
C2–N3–C4	119.38(15)	119.64(15)	118.61(19)	118.0(2)	118.14(17)	118.18(18)
N3–C4–C5	121.72(16)	121.43(16)	122.8(2)	123.3(2)	122.75(19)	122.74(19)
N3–C4–N9	126.15(16)	126.06(16)	129.8(2)	129.4(2)	129.90(19)	129.67(19)
C5–C4–N9	112.13(15)	112.50(16)	107.44(19)	107.2(2)	107.35(18)	107.56(18)
C4–C5–C6	123.09(16)	123.37(16)	122.8(2)	122.5(2)	122.24(18)	122.85(19)
C4–C5–N7	104.97(15)	104.82(15)	107.4(2)	107.67(19)	107.15(17)	107.15(17)
C6–C5–N7	131.84(16)	131.80(16)	129.6(2)	129.3(2)	130.48(18)	129.76(18)
N1–C6–C5	111.79(15)	111.74(15)	111.5(2)	111.0(2)	111.58(17)	111.01(18)
C5–N7–C8	105.86(15)	106.14(15)	107.77(19)	107.45(19)	108.17(17)	108.09(17)
N7–C8–N9	113.82(16)	113.47(16)	110.0(2)	110.1(2)	109.99(18)	109.90(18)
C4–N9–C8	103.22(15)	103.07(15)	107.31(19)	107.55(19)	107.33(17)	107.30(17)

Table S2. Bond distances [Å] and angles [°] in compounds **3**·2DMSO, (*P*)-**3**·(*M*)-3H_2O, and (*M*)-**3**·(*P*)-3H_2O.

	**3**·2DMSO		(*P*)-**3**·(*M*)-3H_2O	(*M*)-**3**·(*P*)-3H_2O
	ring a	ring b		
Ag1–C8	2.083(3)	2.081(3)	2.065(14)	2.065(14)
Ag1–O11	2.137(2)	2.156(2)	2.130(13)	2.129(13)
N1–C2	1.387(4)	1.394(4)	1.385(16)	1.383(17)
N1–C6	1.410(3)	1.404(4)	1.416(15)	1.420(15)
C2–N3	1.394(3)	1.395(4)	1.40(2)	1.41(2)
N3–C4	1.371(3)	1.372(3)	1.378(19)	1.38(2)
C4–C5	1.362(4)	1.364(4)	1.360(18)	1.356(18)
C4–N9	1.367(3)	1.370(3)	1.382(19)	1.385(19)
C5–C6	1.432(4)	1.435(4)	1.436(16)	1.432(16)
C5–N7	1.388(3)	1.385(3)	1.386(18)	1.378(19)
N7–C8	1.338(3)	1.345(4)	1.352(15)	1.355(15)
C8–N9	1.378(3)	1.382(3)	1.37(2)	1.37(2)
C8–Ag1–O11	170.78(10)	170.42(10)	166.7(5)	166.4(5)
C2–N1–C6	126.7(2)	126.6(2)	128.4(11)	128.6(11)
N1–C2–N3	117.8(2)	117.6(2)	116.6(12)	116.3(12)
C2–N3–C4	118.2(2)	118.7(2)	119.0(11)	118.8(11)
N3–C4–C5	122.8(2)	122.4(2)	121.9(12)	122.0(13)
N3–C4–N9	129.9(2)	130.2(3)	130.1(13)	129.7(13)
C5–C4–N9	107.3(2)	107.4(2)	108.0(13)	108.1(13)
C4–C5–C6	123.0(2)	122.9(2)	124.1(12)	124.4(12)
C4–C5–N7	106.6(2)	106.6(2)	105.6(11)	105.4(12)
C6–C5–N7	130.4(2)	130.5(3)	130.1(11)	130.1(11)
N1–C6–C5	111.3(2)	111.7(2)	109.8(10)	109.7(10)
C5–N7–C8	110.5(2)	110.8(2)	111.7(12)	112.2(12)
N7–C8–N9	105.7(2)	105.3(2)	104.9(12)	104.6(12)
C4–N9–C8	109.9(2)	109.9(2)	109.7(11)	109.7(11)
